# Distinct roles of haspin in stem cell division and male gametogenesis

**DOI:** 10.1038/s41598-021-99307-8

**Published:** 2021-10-06

**Authors:** Katerina Soupsana, Eleftheria Karanika, Fani Kiosse, Anastasia Christogianni, Yiorgos Sfikas, Pantelis Topalis, Anna Batistatou, Zoi Kanaki, Apostolos Klinakis, Anastasia S. Politou, Spyros Georgatos

**Affiliations:** 1 Stem Cell and Chromatin Group, The Institute of Molecular Biology and Biotechnology, Biomedical Division, FORTH-ITE, Ioannina, Greece; 2 The Laboratory of Biology, University of Ioannina, Faculty of Medicine, Ioannina, Greece; 3 The Laboratory of Biological Chemistry, University of Ioannina, Faculty of Medicine, Ioannina, Greece; 4 The Department of Computer Science and Engineering, University of Ioannina, Ioannina, Greece; 5 Institute of Molecular Biology and Biotechnology, FORTH, Heraklion, Greece; 6 The Laboratory of Pathology, University of Ioannina, Faculty of Medicine, Ioannina, Greece; 7 The Biomedical Research Foundation, Academy of Athens, Athens, Greece; 8 University Research Center of Ioannina, Institute of Biosciences, Ioannina, Greece

**Keywords:** Spermatogenesis, Embryonic stem cells, Mitosis

## Abstract

The kinase haspin phosphorylates histone H3 at threonine-3 (H3T3ph) during mitosis. H3T3ph provides a docking site for the Chromosomal Passenger Complex at the centromere, enabling correction of erratic microtubule-chromosome contacts. Although this mechanism is operational in all dividing cells, haspin-null mice do not exhibit developmental anomalies, apart from aberrant testis architecture. Investigating this problem, we show here that mouse embryonic stem cells that lack or overexpress haspin, albeit prone to chromosome misalignment during metaphase, can still divide, expand and differentiate. RNA sequencing reveals that haspin dosage affects severely the expression levels of several genes that are involved in male gametogenesis. Consistent with a role in testis-specific expression, H3T3ph is detected not only in mitotic spermatogonia and meiotic spermatocytes, but also in non-dividing cells, such as haploid spermatids. Similarly to somatic cells, the mark is erased in the end of meiotic divisions, but re-installed during spermatid maturation, subsequent to methylation of histone H3 at lysine-4 (H3K4me_3_) and arginine-8 (H3R8me_2_). These serial modifications are particularly enriched in chromatin domains containing histone H3 trimethylated at lysine-27 (H3K27me_3_), but devoid of histone H3 trimethylated at lysine-9 (H3K9me_3_). The unique spatio-temporal pattern of histone H3 modifications implicates haspin in the epigenetic control of spermiogenesis.

## Introduction

Haspin was originally identified in mouse spermatids^1^. Subsequent studies revealed its presence in somatic cells, albeit at much lower levels^2,3^. The carboxy-terminal region of this protein shows homology to a variety of eukaryotic protein kinases^2,4^, possesses a bilobar fold and can constitutively phosphorylate protein substrates^5–8^. Due to the lack of characteristic motifs present in other protein kinases, haspin is classified as an *atypical* serine-threonine kinase. The single haspin gene, *Gsg2*, is also atypical: it lacks introns and lies entirely within an intron of the Integrin alphaE gene^4,9^. Orthologs of *Gsg2* exist in many eukaryotic species, from humans to budding yeast^5^.

Three bibliographical milestones have shaped the current understanding with regards to the function of haspin. In 2004, we have described the reversible phosphorylation of threonine-3 in histone H3 (H3T3ph), a post-translational modification that occurs specifically during mitosis in somatic cells. H3T3ph was localized in the centromeric region of metaphase chromosomes, suggesting a role in chromosome congression^10^. In 2005, Higgins and co-workers showed that the H3T3ph mark is established by haspin^11^. Finally, in 2010 and 2011, several laboratories showed independently that H3T3ph provides a binding site for survivin, a component of the Chromosomal Passenger Complex (CPC)^12–15^. Apart from survivin, CPC also contains INCENP, borealin and the mitotic kinase Aurora B^16^.

Consistent with a role in CPC recruitment, treatment of somatic cells with haspin-specific inhibitors or knockdown with RNAi causes mobilization of Aurora B from the centromere and partial dispersion to the chromosome arms^11,17–20^. Dissociation from the inner centromere compromises chromosome congression, because Aurora B is required for de-stabilization of inappropriate chromosome-microtubule contacts and activation of the spindle assembly checkpoint (SAC)^21^. Apart from chromosome congression, haspin has also been implicated in sister-chromatid cohesion^22,23^ and spindle pole assembly^24^.

On a system’s level, two questions about haspin are still pending. First, given its role in mitosis, why haspin shows its highest expression in haploid spermatids, a non-dividing lineage^1^? Second, since multiple cycles of mitotic division are required for expansion of embryonic cell populations during development, how do we explain that haspin-null embryos develop normally and do not exhibit anatomical defects, apart from testicular anomalies^25^?

Urged by these questions, we set to examine whether haspin has multiple roles or is primarily involved in a tissue-specific function. Using the experimental platform of mouse embryonic stem cells and exploiting the unique spatio-temporal pattern of gametocyte differentiation in the seminiferous tubules of the testis, we have reached the conclusion that haspin is dispensable for completion of mitosis, but likely participates in the transcriptional regulation of spermiogenesis.

## Results

### Haspin dosage affects the levels and the spread of H3T3ph across chromosomes

**Figure 1:**
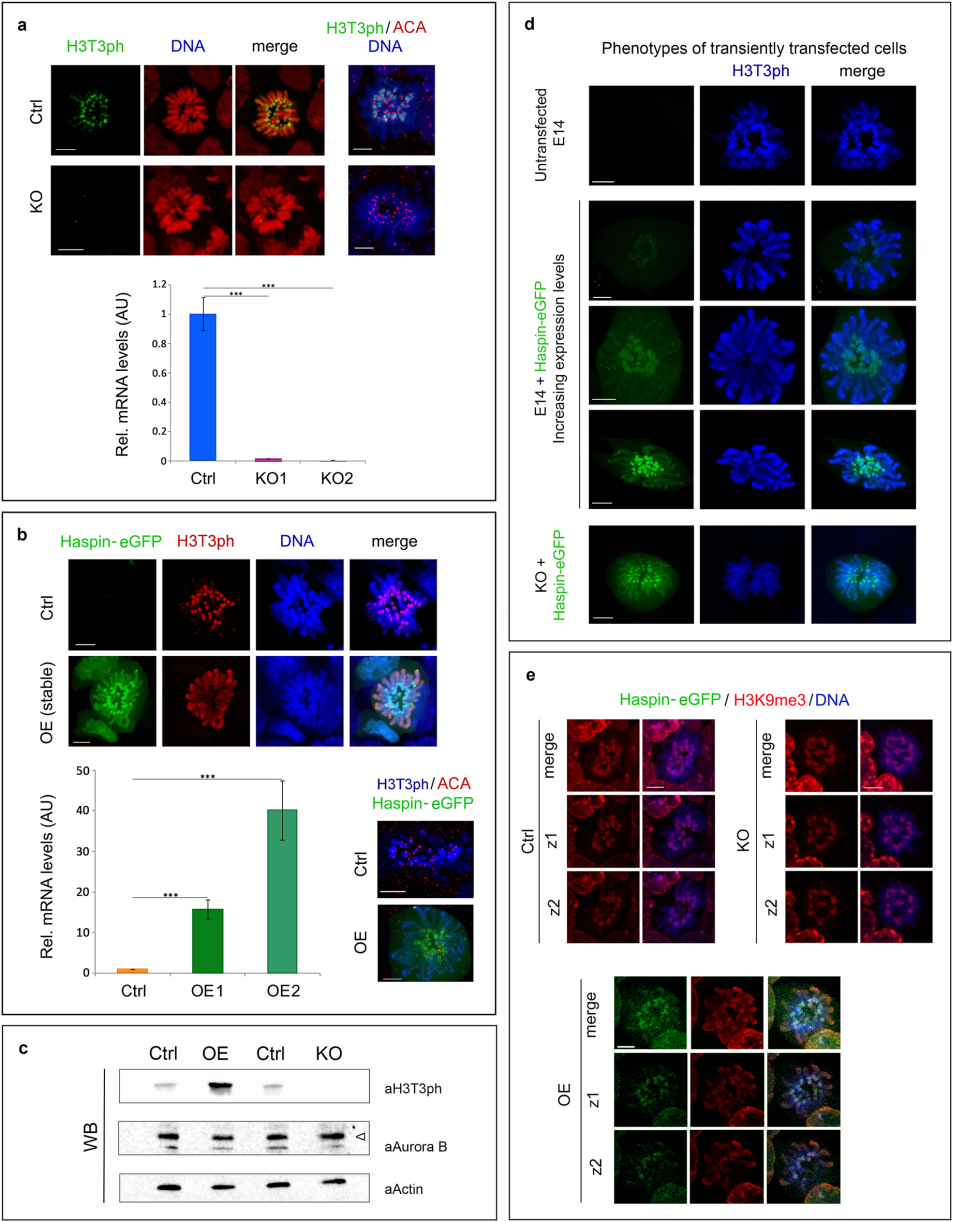
Haspin knockout and overexpression. Occurrence of H3T3ph (images) and levels of haspin mRNA (histograms) in haspin-ΚΟ (**a**) and haspin-ΟΕ (**b**) cells, as detected by indirect immunofluorescence and RT-qPCR assays, respectively. (**c**) Western blots with similar samples. The antibodies used recognize H3T3ph, centromeric antigens (ACA), Aurora B and actin (a loading control), as indicated. For full length blots, see Supplemental Data III. (**d**) Distribution of H3T3ph and haspin-eGFP in transiently transfected E14 cells expressing different levels of the fusion protein. The bottom row shows transiently transfected haspin-KO cells expressing exogenous haspin. (**e**) Spatial distribution of H3K9me_3_ in haspin-KO and haspin-OE cells. Merges and different confocal sections (z1 and z2) are shown. (Ctrl) corresponds to control cells. In all images, DNA has been stained with TO-PRO 3. Scale bars, 5 μm. RT-qPCR data with one of the two KO clones (KO1) has also been presented in a previous publication^20^.

To find out whether haspin has an essential role in self-renewal divisions, we disrupted the *Gsg2* gene using CRISPR/Cas9 genome editing (Fig. 1a, histogram and Supplemental Figure S1; see also 20). In parallel, we generated stable E14 cell lines overexpressing haspin-eGFP (Fig. 1b, histogram). Haspin-knockout (KO) and haspin-overexpressing (OE) cells were examined exhaustively to pinpoint potential mitotic defects.

Analysis of total lysates of KO cells by western blotting revealed the absence of H3T3ph (Fig. 1c, WB, lane KO). Furthermore, no trace of H3T3ph could be detected when these cells were surveyed by confocal microscopy (Fig. 1a, images). Despite the absence of H3T3ph, mitotic chromosomes possessed compact centromeres (as documented by ACA staining), had the usual density and were organized as a normal-looking metaphase plate. The typical, centromeric pattern of H3T3ph in wild type E14 cells remained unchanged after treatment with a specific inhibitor of the Per-Arnt-Sim kinase (PASK), which has been reported to phosphorylate histone H3 at multiple threonine residues in C2C12 and HEK293T cells (Supplemental Figure S2; for relevant literature see Karakkat et al.^26^**)**. Taken together, these results indicate that haspin is solely responsible for H3T3ph and that the lack of this mark does not affect the microscopic appearance and spatial organization of mitotic chromosomes.

Haspin-eGFP, which was produced at high levels in stable OE cell lines, accumulated primarily in the centromeric regions of metaphase chromosomes (Fig. 1b, images). A similar localization pattern was observed in KO cells and wild type E14 cells expressing low or high levels of haspin after transient transfection (Fig. 1d, Phenotypes). Thus, the accumulation of haspin at the centromeres did not seem to depend on expression levels and did not require an initial “seed” of H3T3ph at the centromere.

As expected, the increased dosage of haspin in OE cells resulted in a significant increase of the H3T3ph levels (Fig. 1c, WB, lane OE). However, while haspin-eGFP accumulated at the centromeres, (the excess) H3T3ph spread to the chromosome arms (Fig. 1b,d, images). The presence of H3T3ph across the entire chromosome is easily explained by the increase of haspin kinase activity, which most likely upsets the normal kinase-phosphatase balance. Consistent with this interpretation, a pan-chromosomal distribution of H3T3ph has been observed previously in human U2OS cells deficient in Repo-Man, the regulatory subunit of PP1γ, which counteracts haspin-mediated phosphorylation^27^. The same pattern has been seen in early human zygotes, where the levels of PP1γ are naturally low^28^.

The spread of H3T3ph along the chromosomes likely affects chromatin organization and chromatid packing. A strong indication for this was that histone H3 trimethylation at lysine-9 (H3K9me_3_), a histone mark normally restricted to the pericentromeric area of the mitotic chromosomes, was clearly delocalized in OE cells (Fig. 1e). Furthermore, the sister-chromatids of these cells sometimes appeared “inflated” or partially dissociated from each other (Supplemental Figure S3).

### Aurora B can be recruited to the centromere by a haspin-independent mechanism

**Figure 2:**
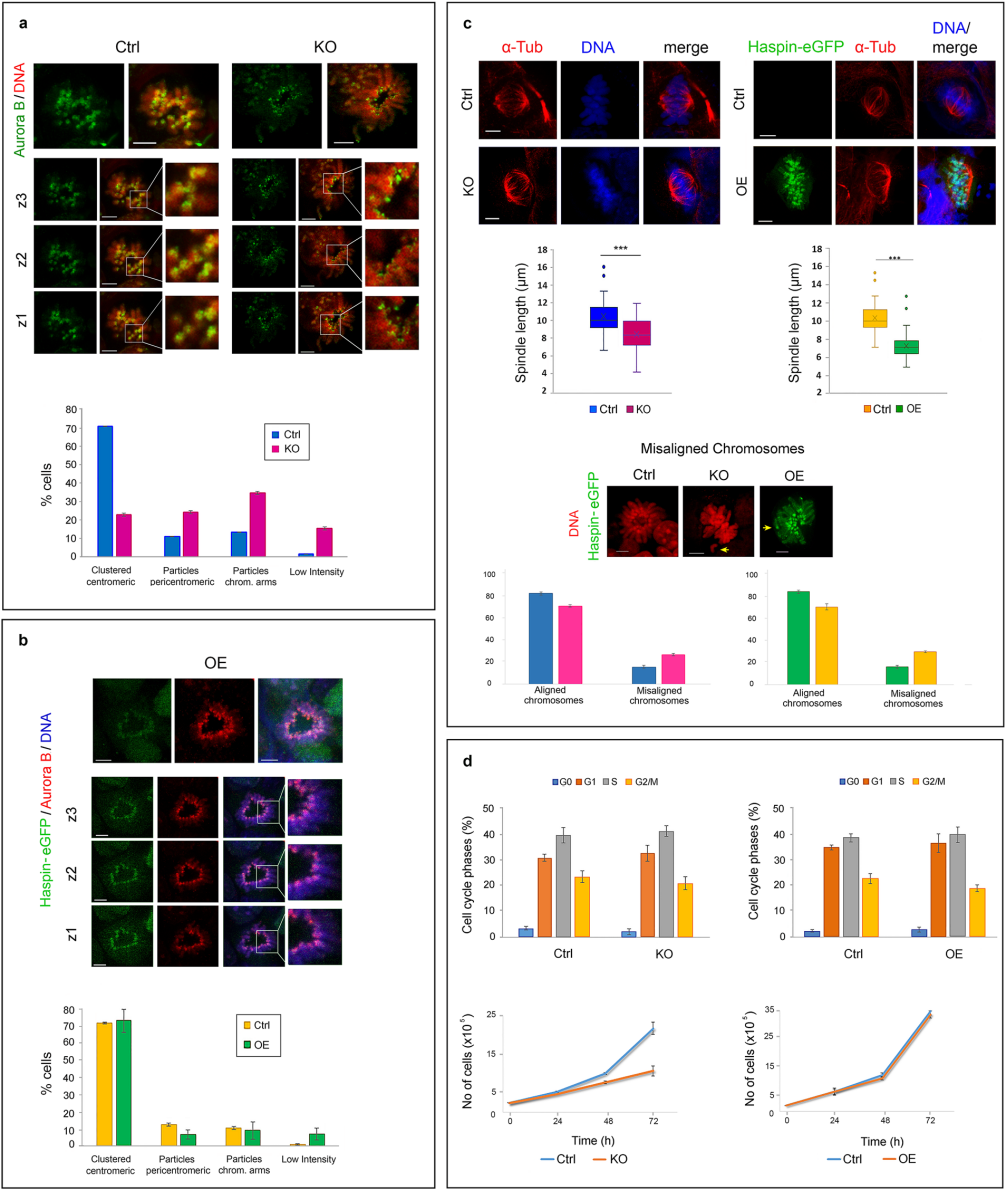
Effects of haspin dosage on cell division and growth. Localization of Aurora B during metaphase in haspin-KO (**a**) and haspin OE (**b**) cells, as detected by indirect immunofluorescence. Maximum projection profiles (top rows) and different confocal sections (z1–z3) are shown in each case. Merged images are also included, together with a contrasted blowup. Histograms display the distribution of Aurora B along mitotic chromosomes in different subsets of cells. The localization patterns of Aurora B are explained in more detail in Figure S4. (**c**) Effects of haspin knockdown or overexpression on spindle length and chromosome congression. The images depict metaphase cells stained with antibodies against α-tubulin and/or DNA. The boxplots display the length of the spindle, whereas histograms show the incidence of misaligned chromosomes in metaphase cells. (**d**) Cell cycle kinetics (histograms) and growth rate (line plots) of haspin-KO and haspin-OE cells. (Ctrl) corresponds to control cells. In all cases, DNA has been stained with TO-PRO 3. Scale bars, 5 μm. For quantitative details and statistical analysis, see Supplementary Data IΙ.

As anticipated from previous studies, in the absence of haspin, the kinase Aurora B was partially de-localized from the centromere (Fig. 2a, upper panel). However, in most of the KO cells, the Aurora B signal appeared weak, as has been previously noted by Baron et al.^29^. A decrease in the steady state levels of Aurora B could be ruled out by western blotting (Fig. 1c, WB, lane KO). Down-regulation of the Aurora B gene could also be excluded by RNA sequencing data presented in a following section. However, the seemingly weak Aurora B signal could be explained by a change in aggregation state.

Closer inspection of KO cells revealed that the large, Aurora B-containing clusters, which were always observed in the centromeric regions of control cells, were replaced by a constellation of small granules. These “fragments” were retained in the pericentromeric zone (Fig. 2a, upper panel, blowups; for more details see Supplemental Figure S4), suggesting H3T3ph-*independent* binding to the proximal centromere/kinetochore^14,30,31^. Corroborating the idea of dual (i.e., H3T3ph-dependent and H3T3ph-independent) binding of CPC to the centromere, Aurora B was predominantly localized at the centromeric region in OE cells (Fig. 2b, lower panel), despite the fact that H3T3ph was spread across the chromosome arms. Finally, supporting a dual mode of CPC/Aurora B-centromere interactions, combined inhibition of the haspin kinase (by 5-ITu) and the Bub1 kinase (by BAY-320) in Hela cells had a more pronounced effect on the targeting of CPC components to the centromere than inhibition of each kinase alone (Supplemental Figure S5a,b; see also Ref.^29^). This experiment could not be performed using E14 cells, because the murine Bub1 kinase was not sensitive to BAY-320; see Supplemental Figure S5c. It is unclear why Aurora B organizes in large centromeric clusters when the haspin-dependent and the haspin-independent mechanisms are both operative, but shows a dispersed distribution when the former mechanism is dismantled.

### Haspin imbalance causes mitotic defects, but does not abort self-renewal divisions

KO and OE cells exhibited mitotic spindle anomalies. In both cases, the inter-pole distance during metaphase was consistently shorter than in the corresponding controls (Fig. 2c), suggesting that correct spindle geometry requires exact haspin dosage. Consistent with the partial de-localization of Aurora B and the altered geometry of the spindle, the incidence of misaligned chromosomes in these cells was increased about two-fold (Fig. 2c, histograms).

Despite the mitotic defects detected in KO and OE cells, the relative proportions of prophase, metaphase, anaphase and telophase figures in the cell population were similar to those of the controls (Supplemental Figure S6a). However, as could be confirmed by video microscopy a proportion of the cells exited mitosis with delayed kinetics (Supplemental Figure S6b,c). No other differences in cell cycle could be detected in the bulk sample by flow cytometry (Fig. 2d, histograms). KO cells (and to a lesser extent OE cells) showed a decrease in growth rate, but the expansion of the self-renewing population was not affected severely (Fig. 2d, growth curves). Taken together, these data show that haspin plays a variety of roles during mitosis, but is clearly not essential for completion of mitosis and population expansion of embryonic stem cells. This interpretation is also justified by the fact that KO and OE cells could be passaged multiple times during selection of the corresponding clones, without a visible change in colony-forming ability.

### Haspin dosage affects heterochromatin organization, but does not compromise the pluripotent state

Recent work has shown that haspin deficiency leads to formation of smaller-than-normal nuclei in *D. melanogaster*^32^. As shown in Supplemental Figure S7, some small (but statistically significant) differences in nuclear size could also be detected in mouse cells that expressed inappropriate amounts of haspin. In this case, the nuclei of KO cells were larger than those of the corresponding controls, while the nuclei of the OE cells were smaller. Although the distribution of heterochromatin protein 1α (HP1α) and histone H3 trimethylated at lysine-9 or lysine-27 (H3K9me_3_ and H3K27me_3_, respectively) did not change, KO and OE cells possessed on the average more heterochromatic foci than the corresponding controls, while the size of the foci in the former was slightly smaller. We interpret these slight (but distinct) differences as indications of altered chromatin organization.

No major effects on the expression of key pluripotency factors, such as Nanog, Oct4 and Klf4, were detected when KO and OE cells were compared to wild type cells by indirect immunofluorescence (Supplemental Figure S8a). Moreover, quantitative assessment of the corresponding mRNAs by qRT-PCR did not reveal consistent differences between KO cells and the corresponding controls (Supplemental Figure S8b, KO). A statistically significant increase in the levels of Nanog, Oct4 and Klf4 mRNA was detected in one of the two OE clones (Supplemental Figure S8b, OE), but since this effect was clearly clone-specific it was not pursued further.

### Haspin is not essential for stem cell differentiation

**Figure 3:**
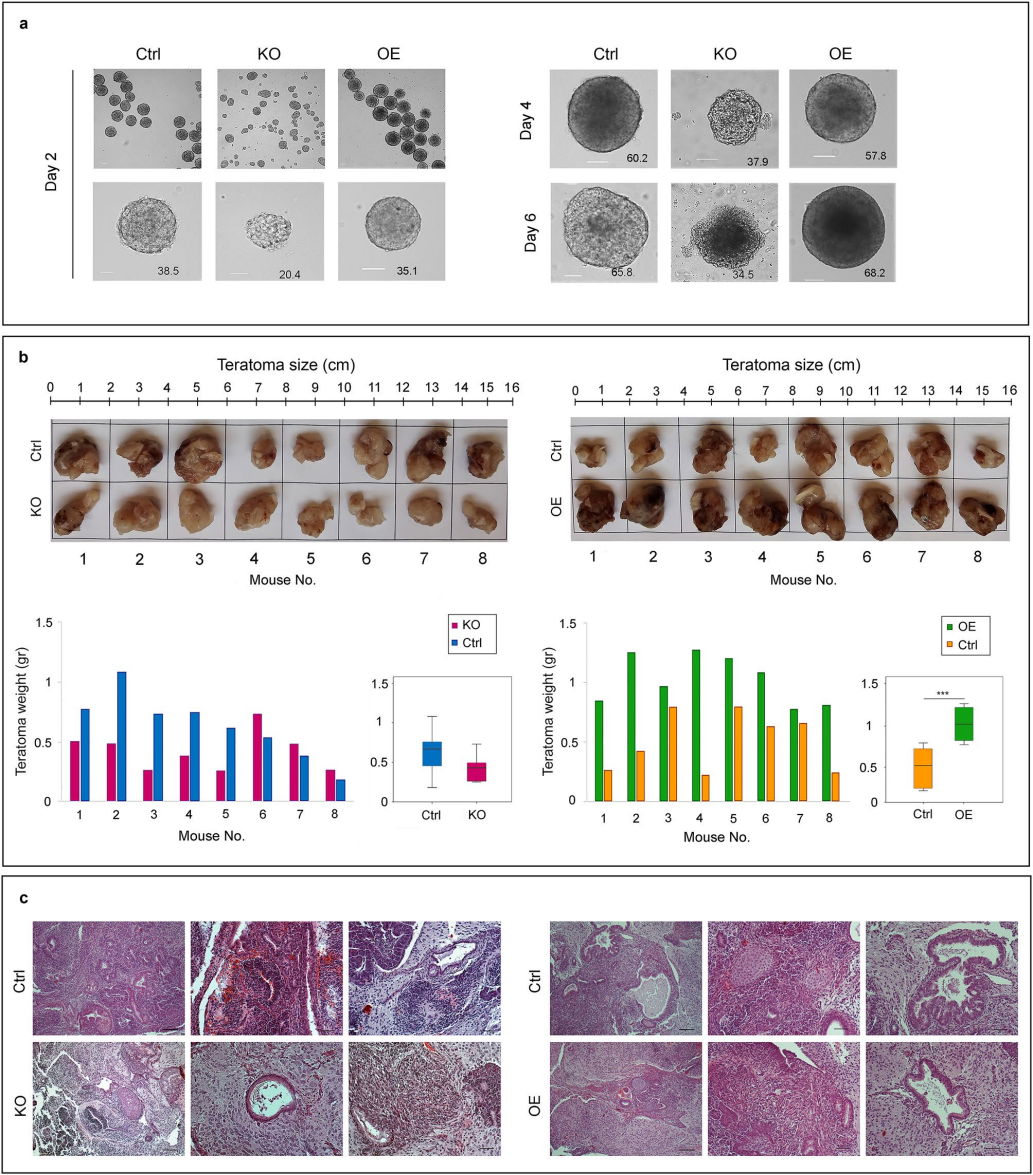
Effects of haspin dosage on differentiation. (**a**) Formation and growth of embryoid bodies with haspin-KO and haspin-OE cells, as visualized by phase-contrast microscopy. The average surface area of embryoid bodies (in μm^2^ × 10^3^) is indicated. Scale bars, 50 μm. (**b**) Teratoma formation by haspin-KO and haspin-OE cells 8 weeks after injection into immunocompromised mice. The relative sizes of the tumors are shown on the images and the corresponding histograms. Boxplots display the weight (in grams) of the teratomas in each ensemble. (**c**) Histological profile of the teratomas. Representative paraffin sections stained with Hematoxylin/Eosin. All three germ layers are present. Visible in the specimens are squamous epithelium (ectoderm); respiratory epithelium (endoderm); neural rosettes or mature neural tissue (ectoderm); and blood vessels (mesoderm). Masses of cartilage and muscle tissue (mesoderm) can also be distinguished in the teratomas, which also contain neural glial cells (ectoderm). Scale bars, 100 μm.

Upon removal of LIF and suspension in “hanging drops”, KO and OE cells produced embryoid bodies (EBs). The EBs formed by the former were generally smaller than those formed by the latter and the corresponding controls (Fig. 3a, KO). Furthermore, whereas EBs derived from OE cells grew normally the first 6 days in culture, those formed by KO cells failed to thrive (Fig. 3a, OE). This failure to thrive is consistent with the fact that KO cells show a relatively slower growth rate than OE and control cells (consult Fig. 2d).

To examine the developmental potential of KO and OE cells by a different method, we induced the formation of teratomas in immunodeficient mice. As shown in Fig. 3b, the tumors derived from KO cells appeared smaller than those derived from the corresponding controls. However, this difference in size was not statistically significant. On the other hand, teratomas formed by OE cells were significantly larger than those formed by control cells, suggesting a difference in growth potential. Histological analysis of the teratomas revealed the existence of tissues derived from all three germ layers (Fig. 3c), in full agreement with the fact that KO mice develop to term and are born alive^25^. These observations establish the fact that haspin is not essential for stem cell differentiation. However, the data do not rule out the possibility that this kinase is involved in some specialized, tissue-specific function.

### Haspin dosage affects gene expression

**Figure 4:**
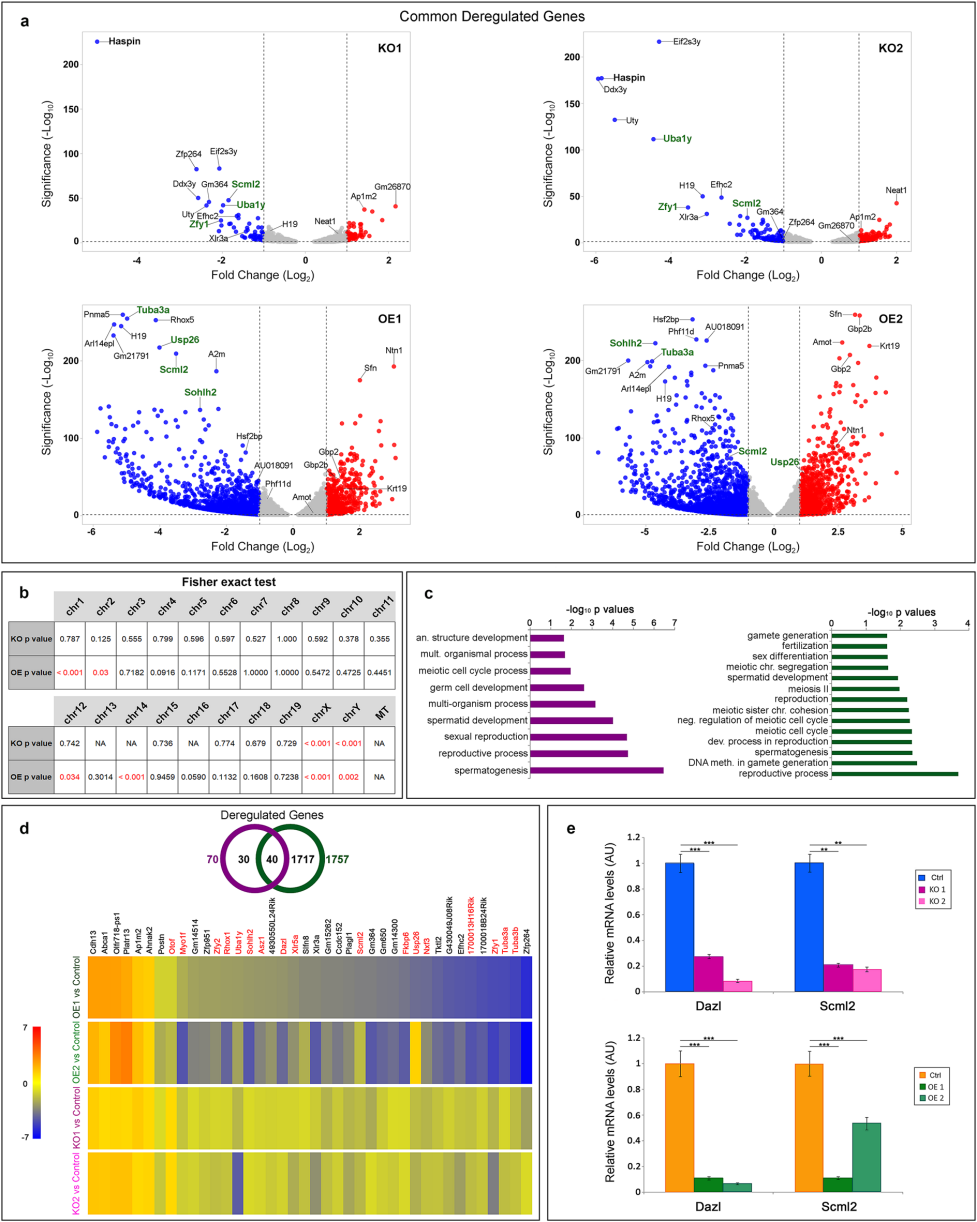
Transcriptome analysis of haspin-KO and haspin-OE cells. (**a**) Volcano plots depicting the fold change and p-values of the deregulated genes that are common in the KO1/KO2 and the OE1/OE2 clones. The horizontal line corresponds to log_10_ ≥ 1.3 and the vertical line to log_2_ ≥ 1. The top 10 hits in each clone, as calculated from their Manhattan distance, are indicated in both plots. Genes involved in gametogenesis are green. (**b**) Correlation between the number of deregulated genes common to KO1/KO2 or OE1/OE2 clones and the total number of genes in each mouse chromosome, as assessed by the Fisher exact test. (**c**) Selected gametogenesis Gene Ontology categories that show statistically significant enrichment (p < 0.05), when deregulated genes common to KO1/KO2 and OE1/OE2 are analyzed. (**d**) Venn diagram displaying the number of genes affected in both haspin-KO and haspin-OE cells and heatmap showing the log_2_ Fold Change of the 40 genes that are common in all clones, irrespective of haspin content. Genes directly or indirectly involved in gametogenesis are in red. (**e**) Relative expression levels of *Dazl* and *Scml2*, as assessed by RT-qPCR.

To determine whether the lack or the excess of haspin influences differentiation along a specific lineage, we analyzed the expression patterns of KO and OE cells by RNA sequencing. As shown in Fig. 4a, a large number of genes were deregulated upon haspin knockout or overexpression. Filtering out those that were affected in only one of the two KO or OE clones, adopting a p-value < 0.05 and concentrating our attention on expression differences ≥ 2-fold over the control, we found that in both cases the downregulated genes outnumbered the upregulated ones. Thus, both the lack and the excess of haspin appeared to have a net repressive effect on gene transcription.

Genes affected by haspin knockout or overexpression could be identified in several mouse chromosomes. According to the Fisher exact test, the distribution of deregulated genes in OE cells exhibited a strong statistical correlation with chromosomes 1, 2, 12, 14, X and Y (Fig. 4b). KO cells yielded a more distinct cytogenetic signature, since the affected genes correlated only with the X and Y chromosomes. Consistent with preferential association with the sex chromosomes, Gene Ontology (GO) analysis indicated that many of the affected genes encoded factors involved in reproductive processes and in particular male gametogenesis (Fig. 4c; consult also Fig. 4a).

To better interpret the genomic data, we focused on a subset of genes, whose expression was significantly changed in *both* KO and OE cells. This category included 40 genes (Fig. 4d). Of these, 33 were downregulated and 6 upregulated in both cases, whereas 1 was differentially expressed in the two groups. The set of downregulated genes included *Scml2* and *Dazl*. *Scml2* (Sex comb οn midleg-like 2) resides in the X chromosome and encodes a Polycomb-associated protein responsible for establishing the male epigenome through histone H2A ubiquitination^33–35^. *Dazl* (Deleted in azoospermia-like) is located at chromosome 17 and encodes an RNA-binding protein that interacts with other RNA-binding proteins to regulate mRNA translation in spermatogonia and primary spermatocytes^36,37^. The downregulation of these two genes was confirmed by qRT-PCR (Fig. 4e).

Downregulated genes also included *Zfy1/2*, two genes located in the Y chromosome and involved in spermatogenesis^38–43^; *Uba1y*, a gene also localized in the Y chromosome and involved in the survival and proliferation of differentiating spermatogonia^44–46^; *Sohlh2*, an essential germ cell-specific gene^47–50^; *Nxf3*, a gene localized in the X chromosome and specifically expressed in Sertoli cells^51,52^; *Fkbp6*, a meiosis-specific gene^53^; and the upstream regulator of the *EIF2S3Y* gene, which, together with *SRY,* orchestrates the male gametogenesis program^54–56^. Finally, among the genes that were downregulated in OE cells were *Zfp264* and *Usp26*. The former gene lies in a reciprocal imprinting domain and is highly expressed in testis^57^; the latter (which was also downregulated in one of the two KO clones) encodes a protein whose mutation results in deficiencies in fertility and spermatogenesis, depending genetic background (Sakai et al.^58^).

Using the STRING algorithm and the database of protein–protein interactions^59^, we found that certain haspin targets are parts of a gene network. As shown diagrammatically in Supplemental Figure S9, central in this network are some of the most downregulated genes in the KO clones (> 6-fold difference; see Fig. 4a), e.g., KDM5D (*JARID1D*) and *Uty*, which encode N^ε^-methyl lysine demethylases. KDM5D demethylates H3K4me_2,3_, whereas UTY is similar to UTX and JMJD3, two well-known H3K27me_3_ demethylases^60,61^.

### H3T3ph occurs in pre-meiotic, meiotic and post-meiotic gametocytes

**Figure 5:**
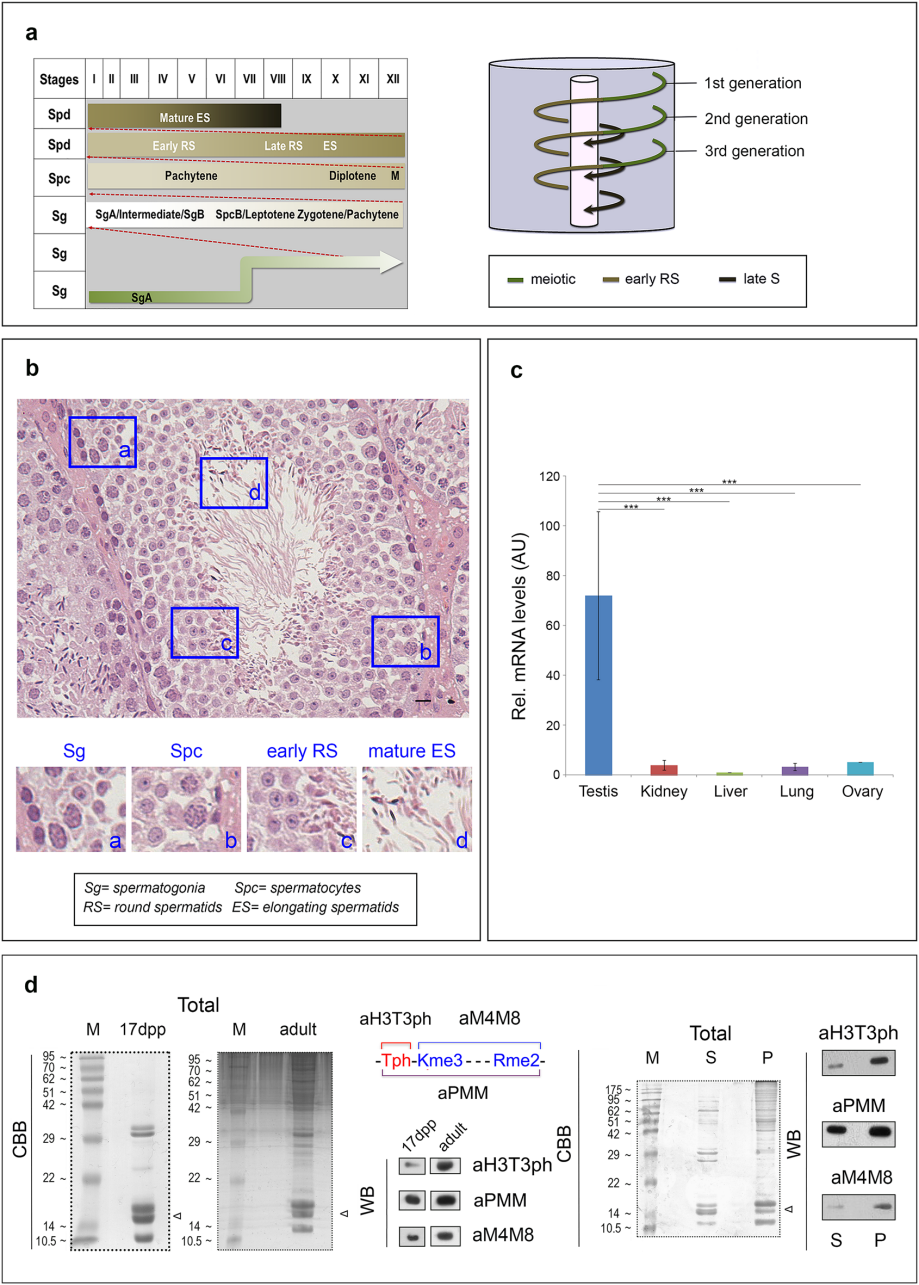
Abundance of haspin and H3T3ph in the mouse testis. (**a**) Schematic representation of the spermatogonial differentiation process (left) and the colonization of the seminiferous epithelium by spermatogenic cells (right). (**b**) A representative paraffin section of adult mouse testis stained with Hematoxylin/Eosin. The different cell types are indicated. Scale bar, 10 μm. (**c**) Relative levels of haspin mRNA in various tissues as assessed by RT-qPCR. (**d**) Western blotting (WB) experiments using the aH3T3ph, aPMM and aM4M8 antibodies (for details see text). The permutations of the PMM motif (H3T3phK4me_3_, H3K4me_3_R8me_2_, H3T3phR8me_2_) are shown graphically on top of the second panel. Total histones from immature (17-day old) and adult mouse testes were extracted by HCl and analyzed by SDS-PAGE/Coomassie brilliant blue (CBB) staining, as indicated. Portions of this material were used for western blotting. (*M*): molecular mass markers; (*Total*): unfractionated HCl extract; (*S*) supernatant after high speed centrifugation; (*P*): residue after centrifugation. The position of histone H3 is indicated by an arrowhead. For full length gels and blots, see Supplemental Data III.

Prompted by the RNA sequencing data, we focused our attention on male gametogenesis. As shown schematically in Fig. 5a, the male gametogenesis program in the mouse involves a sequence of mitotic and meiotic divisions, via which the undifferentiated, diploid spermatogonia are converted to haploid spermatids and, eventually, to mature spermatozoa^62–64^. A typical histological profile of the seminiferous tubule in the mouse testis is shown in Fig. 5b.

Using RT-qPCR, we found that haspin transcripts are far more abundant in testes than in other organs (Fig. 5c). This agrees fully with the pioneering work of Tanaka et al.^1^, who showed that haspin is particularly enriched in haploid spermatids, and is in line with more recent transcriptome data, which show that expression of the *Gsg2* gene is highly increased at the onset of spermiogenesis^65^, exactly when the expression of several spermatonesis-specific genes declines (see Supplementary Figure S10).

We reasoned that, if haspin is involved in transcriptional regulation during spermiogenesis, it might exert its regulatory role through H3T3ph. To examine the occurrence of H3T3ph in testis, we used two anti-peptide antibodies, aH3T3ph and aPMM, which recognize either H3T3ph or the combinatorial modification signature H3T3phK4me_3_R8me_2_ (PMM). These antibodies have been thoroughly characterized in previous studies^8,10^. For confirmatory work, we also used aM4M8, an antibody raised against the non-phosphorylated, doubly methylated peptide H3T3K4me3R8me2^8^. As shown in Fig. 5d, the three antibodies recognized modified histone H3 in total (acid) extracts of mouse testis and clarified supernatants containing soluble histones.

**Figure 6:**
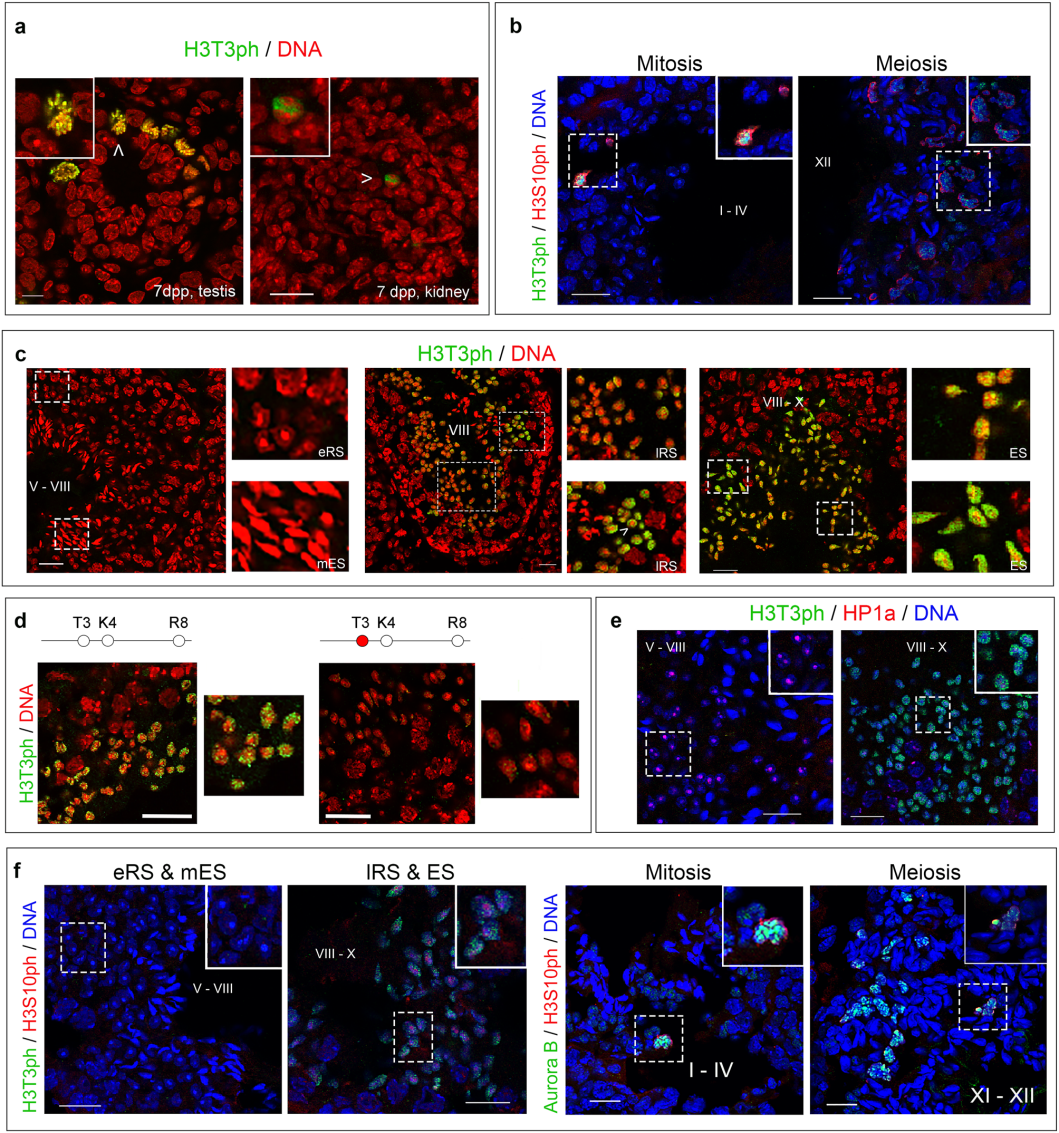
Identification of H3T3ph in testicular cells. (**a**) Frozen sections of immature (7-day old) testis and kidney stained with aH3T3ph. H3T3ph is detected exclusively in mitotic spermatogonia and mitotic kidney cells (arrowheads). Scale bars, 10 μm. (**b**) Staining with aH3T3ph and aH3S10ph antibodies confirmed that both of these modifications are present in mitotically and meiotically dividing cells of the male germline. Scale bars, 20 μm. (**c**) Frozen sections of adult mouse testis depicting seminiferous tubules at different stages of the spermatogenic cycle (Latin numbers). Early round spermatids (eRS) and mature, elongated ones (mES) are shown on the left. Late round spermatids (lRS) are shown in the middle; and elongating spermatids (ES) are shown on the right. Enlarged images show details in each case. Note that in stages V–VIII tubules (left panel) no H3T3ph is detected. The arrowhead points to a chromocenter. Scale bars, 20 μm. (**d**) Competitive inhibition of aH3T3ph staining using the unmodified histone H3 peptide (H3T3K4R8) or the immunogenic (H3T3phK4R8) peptide to confirm specificity. Scale bars, 20 μm. The stick diagram indicates the modifications in residues 3–8 of histone H3, with the H3T3ph mark in red. (**e**) Double staining of stage V–VIII and VIII–X tubules with aH3T3ph and anti-HP1α antibodies. Insets correspond to groups of early round spermatids and late round/elongating spermatids, respectively. Scale bars, 20 μm. (**f**) Double staining with aH3T3ph/aH3S10ph and anti-Aurora B/H3S10ph antibodies during mitosis (spermatogonia), meiosis (spermatocytes) and interphase (spermatids). H3S10ph is present in the chromocenters of late round and elongating spermatids (left) and in the chromosomes of dividing cells, along with Aurora B (right). Scale bars, 20 μm. In all panels, DNA has been stained with TO-PRO 3. For details see text.

The immunoreactive material detected in western blotting experiments was generally less abundant in testes removed from sexually immature mice (17dpp) than in testes obtained from sexually mature animals. However, the most pronounced difference was seen when we used the aH3T3ph antibody (Fig. 5d). Consistent with these data, in frozen sections from 7-day old mouse testis, aH3T3ph stained a rare sub-population of cells residing in the periphery of the seminiferous tubule and corresponding to mitotic spermatogonia (Fig. 6a). In agreement to that and in line with previous studies, aH3T3ph stained exclusively mitotic cells in somatic tissues (e.g., kidney). However, after attainment of sexual maturity, aH3T3ph decorated a variety of cell types, including meiotic spermatocytes (Fig. 6b and Supplemental Figure S11a). A distinct aH3T3ph signal could also be discerned in round spermatids of stage VIII tubules after spermiation (i.e., release of maturing spermatids from Sertoli cells). This signal became increasingly stronger in late round and elongating spermatids, which were abundantly present in stage VIII–X tubules. However, immature spermatids in stage V–VIII tubules were stained weakly or not stained at all (Fig. 6c).

To prove beyond doubt that the antigen recognized in haploid spermatids was indeed H3T3ph, we repeated the immunofluorescence experiments mixing aH3T3ph with either the unmodified peptide H3T3K4R8 or the immunogenic peptide H3T3phK4R8 that was used to raise the aH3T3ph antibody. As shown in Fig. 6d, the signal of H3T3ph was not suppressed by the unmodified peptide, but was virtually abolished by the immunogenic peptide. These data substantiate the existence of H3T3ph in haploid spermatids and further show that this mark correlates with spermatid maturation.

Looking more closely into the sub-nuclear distribution of H3T3ph, we noticed that the mark was excluded from the chromocenter, a dense area of the nucleus that contains constitutive heterochromatin (Fig. 6c, arrowhead). This structure was heavily stained by anti-HP1α antibodies in stage V–VIII tubules, where H3T3ph was scarce, but not in stage VIII–X tubules, which stained heavily with H3T3ph (Fig. 6e). Interestingly, a pattern similar to that of H3T3ph was observed with aH3S10ph, an antibody recognizing histone H3 phosphorylated at serine-10 (see Fig. 6f). The presence of H3S10ph in haploid spermatids was as unexpected as the presence of H3T3ph, because this modification also occurs predominantly during cell division^66^.

### Spermatid chromatin is marked sequentially with H3K4me_3_/H3R8me_2_ and H3T3ph

**Figure 7:**
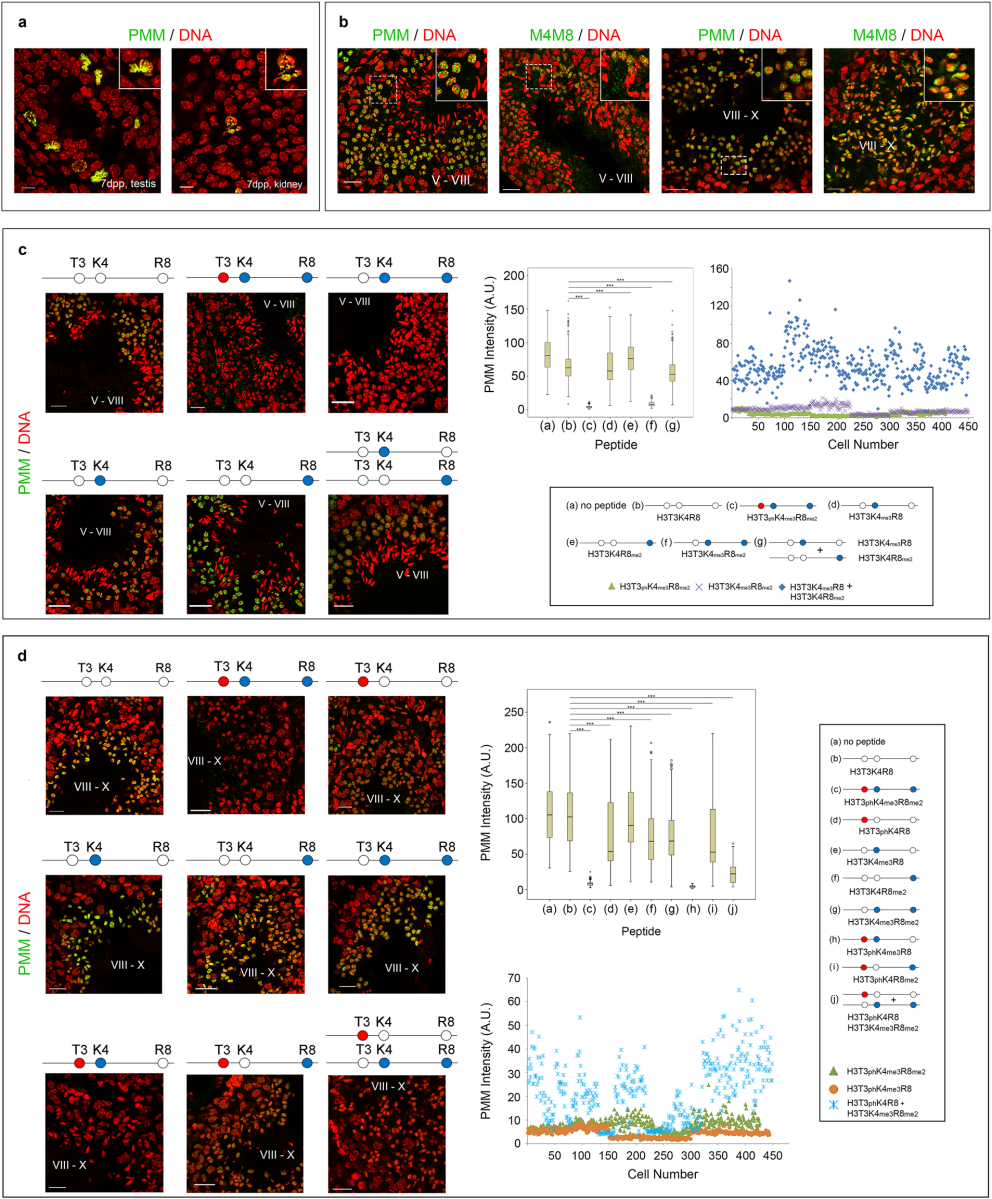
Identification of the PMM signature during the cycle of the seminiferous epithelium. (**a**) Frozen sections of 7-day old mouse testis and kidney stained with aPMM. Insets correspond to mitotic cells. Scale bars, 10 μm. (**b**) Frozen sections of adult mouse testis depicting seminiferous tubules at stages V–VIII and VIII–X stained with aPMM and aM4M8. Scale bars, 20 μm. (**c**) Competitive inhibition assays in stage V–VIII tubules using various synthetic peptides. The red circle in the stick diagram indicates the H3T3ph mark, while the blue circles the H3K4me_3_ and H3R8me_2_ modifications, respectively. Boxplots and scatterplots depict aPMM staining intensity in the presence of the histone H3 peptides. The results confirm the presence of H3K4me_3_R8me_2_ in early round spermatids. Scale bars, 20 μm. (**d**) Competitive inhibition assays in stage VIII–X tubules. Boxplots and scatterplots depict aPMM staining intensity in the presence of the synthetic peptides. The assays confirm the presence of H3T3ph-H3K4me_3_–H3R8me_2_ combinations in late round and elongating spermatids. Scale bars, 20 μm. For quantitative details see Supplementary Data IΙ.

aPMM, the antibody that recognizes the combinatorial modification of histone H3, decorated exclusively mitotic figures in 7-day old mouse testis (Fig. 7a), but stained early round spermatids in stage V–VIII tubules in the adult testis (Fig. 7b, left panel). Since early round spermatids stained very weakly or not at all with aH3T3ph (see Fig. 6c, left and middle panels), we suspected that the antigen recognized in these cells by aPMM was a *part* of the PMM signature, i.e., H3T3K4me3R8me2. This interpretation was further supported by two lines of evidence: first, aPMM was previously found to bind the H3T3K4me3R8me2 peptide in ELISA assays, albeit less avidly than H3T3phK4me_3_R8me_2_^8^; second, early round spermatids in stage V–VIII tubules stained with aM4M8, the anti-peptide antibody raised against the non-phosphorylated, doubly methylated peptide H3T3K4me_3_R8me_2_ (see Fig. 7b).

To prove the point, we performed competition experiments with histone H3 peptides and quantified the results. As shown in Fig. 7c, aPMM staining was affected slightly by the unmodified peptide H3T3K4R8 (20% decrease in average signal intensity), but was virtually abolished by either H3T3phK4me_3_R8me_2_ or H3T3K4me_3_R8me_2_ (> 90% decrease). Much lower inhibition was observed with the singly modified peptides H3T3K4R8me_2_ and H3T3K4me_3_R8 (< 20% decrease), suggesting predominance of the dual methylation mark.

Consistent with the co-existence of the double methylation mark in the same H3 molecule, a *mixture* of H3T3K4me_3_R8 and the H3T3phK4R8me_2_ peptides inhibited aPMM staining to a small extent (30% decrease) by comparison to the inhibition seen with the H3T3K4me_3_R8me_2_ peptide (> 90% decrease). However, when we used the peptide mixture, the variation in the signal intensity of aPMM across the specimen was more pronounced than that observed with the single peptide (see scatterplot in Fig. 7c), which indicates the existence of heterogeneity at the single-cell level.

In stage VIII–X tubules, aPMM stained late round and elongating spermatids (Fig. 7b). Confirming fully our working hypothesis, a modest inhibition was observed in this case with the non-phosphorylated, doubly methylated peptide H3T3K4me_3_R8me_2_ (30% decrease in average signal intensity), but aPMM staining was abolished by H3T3phK4me_3_R8me_2_ and H3T3phK4me_3_R8 (> 90% decrease). The H3T3phK4R8, H3T3phK4R8me_2_ and H3T3K4R8me_2_ peptides inhibited aPMM staining to a lesser extent (~ 30% decrease), while the H3T3K4me3R8 peptide had a negligible effect (5% decrease at this stage).

Competition assays with a *mixture* of H3T3phK4R8 and the H3T3K4me_3_R8me_2_ peptides showed that aPMM staining was in this case significantly inhibited (88% decrease). Again, the scattering of the data points was much more pronounced than in the case where we used the single H3T3phK4me_3_R8me_2_ peptide (see scatterplot in Fig. 7d). Collectively, these results indicated that, although haploid spermatids contain all permutations of the combinatorial signature PMM, they become increasingly richer in H3T3ph and less rich in other modifications upon progression of differentiation.

**Figure 8:**
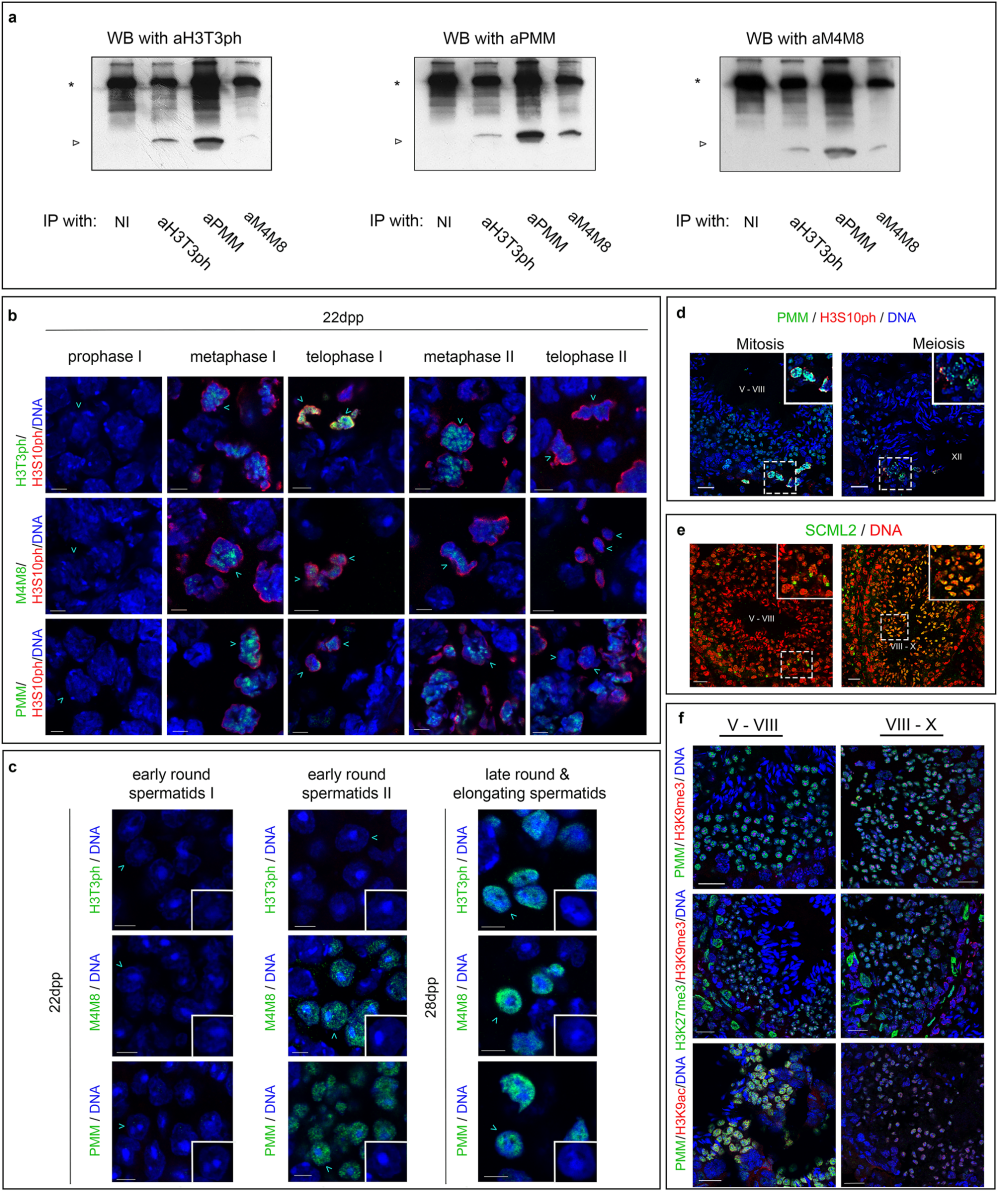
Spectrum of histone H3 modifications during the cycle of the seminiferous epithelium. (**a**) Western blot (WB) and Immunoprecipitation (IP) experiments using the aH3T3ph, aPMM and aM4M8 antibodies. Total histones from adult mouse testes were extracted by HCl and analyzed by SDS-PAGE (see Fig. 5b). Portions of this materials were used for IP assays shown in this figure. An asterisk denoted the heavy chains of IgG and an arrowhead the trace of histone H3. (NI) represents non-immune serum used for control IP. For details see text. Full length blots are presented in Supplemental Data III. (**b**) Detailed representation of the H3T3ph (first row), H3T3K4me_3_R8me_2_ (second row) and PMM marks (third row) during meiotic divisions. Double immunofluorescence experiments with testis sections from 22 day old mice are shown, using H3S10ph as an indicator of dividing cells. DNA has been stained with TO-PRO3. Scale bars, 5 μm. (**c**) Newly formed early round spermatids (eRS I and II) from 22 day old mice, stained with the aH3T3ph, aM4M8 and aPMM antibodies. Profiles of late round and elongating spermatids are also shown. DNA has been stained with TO-PRO3. Scale bars, 5 μm. (**d**) Double immunofluorescence experiments with aPMM and aH3S10ph antibodies. The two marks are detected not only in mitotic spermatogonia (left image, Mitosis), but also in dividing spermatocytes of stage XII tubules (right image, Meiosis). Insets show at higher zoom dividing spermatogonia and spermatocytes carrying the PMM and H3S10ph marks. Scale bars, 20 μm. (**e**) Sections of adult mouse testis stained with an antibody against SCML2. Confocal images confirm the presence of this protein on the XY body of pachytene spermatocytes (left) and on under-condensed regions of the nucleus in late round and elongating spermatids (right). Scale bars, 20 μm. (**f**) Frozen sections of adult mouse testis stained with aPMM/anti-H3K9me3; anti-H3K9me3/anti-H3K27me3; and aPMM/anti-H3K9ac. Scale bars, 20 μm. In all specimens, DNA has been stained with TO-PRO 3.

The co-existence of phosphorylation and methylation marks in *cis* was also investigated using biochemical methods. In these experiments, we combined immunoprecipitation (IP) and western blotting (WB) to examine the modification status of histone H3 extracted from mouse testes by HCl. As shown in Fig. 8a, aH3T3ph reacted strongly with the material precipitated by aPMM and very weakly with the histone H3 fraction precipitated by aM4M8. On the other hand, aPMM reacted strongly with the material precipitated by aM4M8 and weakly with histone H3 precipitated by aH3T3ph. Finally, aM4M8 reacted strongly with the H3 fraction precipitated by aPMM and weakly with the material precipitated by aH3T3ph. Although quantitative conclusions could not be drawn from these assays (because the three antibodies reacted to a different degree with the total H3 complement; see Fig. 5b), it would be safe to conclude that the histone sample contained H3T3ph and to a lesser extent H3T3phK4me_3_ (detected by aH3T3ph), as well as H3TphK4me_3_R8me_2_ and H3K4me_3_R8me_2_ (detected by aPMM), as expected from the peptide-inhibition data shown in Fig. 7c,d.

Combining these observations, we exploited the unique spatio-temporal pattern of differentiation across and along the seminiferous tubule and proceeded in the definitive experiment: to investigate the sequence of events leading to histone H3 modifications as a function of differentiation. For this task, we examined in detail testicular sections from 22 day-old mice, which had not yet completed the first cycle of spermatogenesis. As shown in Fig. 8b, during meiosis I and II both aH3T3ph and aPMM stained the chromosomes, in perfect agreement to previous observations in dividing somatic (Hela) cells^8^. Staining was also observed throughout with the aM4M8 antibody. Early forms of round spermatids were not stained by any of the three antibodies. However, in the transition from one stage of early round spermatids to the next, the aM4M8 and the aPMM antibodies yielded intense staining. The process was completed in late round spermatids, which became positive for H3T3ph. These spermatid forms were identified in 28 day-old mice (Fig. 8c). The data described above provide compelling evidence for sequential modification of histone H3 during spermiogenesis. A summary view of the appearance and disappearance of the H3T3ph and the PMM marks during the seminiferous cycle of adult mice is provided in Supplemental Figure S12.

### The PMM mark occurs simultaneously with H3K27me_3_ in cells expressing SCML2 and DAZL

In sections of adult testis, aPMM decorated mitotic and meiotic figures, which were also positive with antibodies to H3S10ph (Fig. 8d). Antibodies to SCML2, an epigenetic factor affected in KO and OE cells (see Fig. 4), stained the sex body of pachytene spermatocytes in stage V–VIII tubules (Fig. 8e, left panel), as has been previously reported by Hasegawa and co-workers^33^. However, staining of moderately condensed chromatin was also detected in late round and elongating spermatids in stage VIII–X tubules (Fig. 8e, right panel). This pattern was observed only when fixation of the samples was sufficient (4% formaldehyde). Thus, the SCML2 protein should be either less abundant or more labile during spermiogenesis than during spermatogenesis, in agreement with other data, showing that SCML2 acts prior to switching from the spermatogenesis to the spermiogenesis program during differentiation of the male germline^33^. Antibodies to DAZL, another regulatory factor affected in KO and OE cells (see Fig. 4), showed heavy cytoplasmic staining in immature spermatogonia during the first wave of spermatogenesis (Supplemental Figure S11b). However, in the adult testis, the antibodies stained the nuclei of late round and elongating spermatids, which were also decorated by aH3T3ph.

Double staining with aPMM and antibodies to H3 trimethylated at lysine-9 (H3K9me_3_) revealed reciprocal staining of the nucleus in stage V–VIII and VIII–X tubules, with H3K9me_3_ localizing at the chromocenter and PMM distributing in areas of moderately compacted chromatin (Fig. 8f, top panel). PMM-marked chromatin was also decorated with antibodies to H3 trimethylated at lysine-27 (H3K27me_3_; see Fig. 8f, middle panel), but whether the two signals exactly co-localize could not be assessed with certainty using the available antibodies (both were raised in rabbits). Finally, double staining with aPMM and antibodies to H3 acetylated at lysine-9 (H3K9ac) indicated that the two marks coincide in stage V–VIII and VIII–X tubules (Fig. 8f, bottom panel). These data indicate that the PMM signature is present in regions of moderately compacted chromatin and excluded from areas of constitutive heterochromatin.

## Discussion

We have shown here that haspin, although important for mitotic fidelity, is not essential for self-renewal divisions and differentiation of embryonic stem cells. The lack of haspin is probably compensated during mitosis by Bub1 kinase, which phosphorylates histone H2A at threonine-120 (H2AT120ph), creating a binding site for shugoshin (Sgo). Sgo associates with borealin, thus anchoring CPC at the proximal centromere/kinetochore region^14,30,67–69^.

In addition to its function in mitosis, haspin appears to be involved in the transcriptional control of male gametogenesis, thus serving two mechanistically unrelated functions in dividing and in non-dividing cells of the testis, behaving in essence as a “moonlighting protein”^70^. Since male gametogenesis involves both mitotic/meiotic divisions and switching from the soma-specific to the testis specific gene expression program, the role of haspin in this process seems to be important under physiological conditions, even if other factors could partially compensate for the lack of it. That might justify the fact that this protein is (naturally) much more abundant in testis than in other tissues (Tanaka et al.^4^ and data presented here).

The simplest method to explain the involvement of haspin in transcriptional regulation is to assume that H3T3ph is part of a histone modification (sub) code. That both the absence *and* the excess of haspin result in downregulation of several male germline genes cannot be explained by invoking a simple, binary phospho-methyl switch (e.g., H3T3ph-K4me_3_). If this were the case, genes downregulated in haspin OE cells should have been upregulated in KO cells. However, our findings can be readily explained by a mechanism that involves a *compound* phospho-methyl switch (e.g., H3T3ph-K4me_3_-R8me_2_), acting alone or in combination with bivalent histone modifications^35,71^ (e.g., H3K4me_3_-K27me_3_), which do not involve H3T3ph. This is graphically shown in the provisional model of Supplemental Figure S13. This model not only explains why both the absence and the excess of haspin result in gene downregulation, but also why haspin overexpression has a more severe effect on transcription than haspin knockout.

The co-existence of H3T3ph, H3K4me_3_ and H3R8me_2_ *in cis* was reported originally by Markaki et al.^8^, analyzing mono-nucleosomal fractions from mitotic Hela cells by mass spectrometry. Another mass spectrometry study has also shown that histone H3 isolated from sorted populations of testicular cells contains H3T3phK4me_3_^72^. Together with the data reported here, these studies provide compelling evidence for tandem modifications in the histone H3 tail. This is somewhat unexpected because phosphorylation at threonine-3 is known to interfere with trimethylation at lysine-4^6,12,73^. However, the co-existence of mutually interfering and functionally antagonistic modifications is not unprecedented. For example, H3S10ph occurs transiently together with H3K9me_3_^74,75^.

H3K4me_3_ is considered an activating histone modification, because it occurs in the promoter regions of transcribed genes^76,77^. On the other hand, H3R8me_2_ is known to repress transcription in a variety of cellular systems^78–81^. By that measure, the signature H3T3phK4me_3_R8me_2_ might contain a “GO” (K4me_3_), a “STOP” (R8me_2_) and a “RELAY” (T3ph) signal that function as a compound phospho-methyl switch.

A large body of structural and biochemical data justify this idea. The negative effect of H3T3ph on H3K4me_3_ binding has been experimentally proven. For instance, H3T3ph reduces binding of the double chromodomain of the ATP-dependent chromatin-remodeling factor CHD1 to H3K4me_3_ approximately 25-fold by eliminating an intra-peptide bond between threonine-3 and glutamine-5^82^. Similar effects have been reported with regards to the binding of PHD finger proteins (e.g., DIDO3, ING2, MLL5, RAG2 and TAF3) and the TTD module of the lysine-specific demethylase 4A to H3K4me_3_^83–87^.

## Materials and methods

### Cell culture and generation of stable lines

E14 cells were generously provided by Austin Smith’s lab (Cambridge University, UK). They were cultured in Glasgow Minimum Essential Medium (GMEM, Gibco) on plates coated with 0.1% gelatin (Gelatin from bovine skin, Sigma-Aldrich). The medium was supplemented with 15% fetal bovine serum (FBS) (Biochrom AG), 2 mM penicillin/streptomycin (Gibco), 2 mM l-glutamine (Gibco), 0.1 mM non-essential amino acids (Gibco), 1 mM sodium pyruvate (Gibco), 0.1 mM β-mercaptoethanol (Sigma-Aldrich) and LIF (produced in house).

Hela cells were cultured in Dulbecco’s Modified Eagle’s Medium (DMEM) (Gibco) supplemented with 10% fetal bovine serum (Biosera), 2 mM penicillin–streptomycin (Biosera) and 2 mM l-glutamine (Biosera). All cell lines were maintained at 37 °C in a humidified incubator supplemented with 5% CO_2_.

Differentiation assays were performed using the hanging drop method as described previously in Karanika et al.^20^.

Five-iodotubercidin (5-ITu) from Santa Cruz Biotechnology (sc-3531A) was used for haspin inhibition; BAY-320 (BAYER) for Bub1 inhibition; and BioE-1115 (Millipore) was used for inhibition of the PAS kinase. For exact concentrations used see main text.

For transient transfection assays, episomal plasmid pPyCAGIP carrying haspin-eGFP (20 µg) was introduced into cultured cells by electroporation using an ECM630 apparatus (BTX). 5 × 10^6^ Ε14 cells were resuspended in 400 μl full medium, introduced into a 4-cm electrode gap cuvette and porated at 200 V and 950 µF, 0 Ω. To generate cell lines overexpressing haspin (OE), E14 cells were first transfected as above and 24 h later the standard medium was replaced with one containing 1.5 µg/ml puromycin. Selection was carried out for approximately 2 weeks, until visible healthy colonies appeared. Subsequently, single colonies were picked and transferred into separate wells of a 96-well plate containing 0.5 µg/ml puromycin. When wells reached 80–90% confluency, cells were transferred into a 24-well plate containing 0.5 µg/ml puromycin and subsequently into a 12-well plate containing 0.75 µg/ml puromycin. Two additional passages followed, one in a 6-well plate containing 1 µg/ml puromycin and the last one in a 60 mm plate containing 1.5 µg/ml puromycin.

Haspin-knockout lines were produced as described in Karanika et al.^20^.

### Teratoma formation

The teratoma formation assays were carried out in the laboratory of Dr. Klinakis (Academy of Athens-BRFAA, ethics approval ref. number 6245, ARRIVE guidelines compliant) and were approved by the Directorate for Agricultural Economy and Veterinary Services, Prefecture of Attica, Greece. Initially, the cells were trypsinized, to produce a single cell suspension and counted. Then, 10^6^ cells were transferred into a new tube, centrifuged at 500*g* for 3 min and resuspended in 0.1 ml PBS. The cell suspension was injected subcutaneously into the thigh of immunodeficient SCID mice using the following scheme: The left thigh of each mouse was used for KO or OE cells and the right for control cells. For each combination, 8 adult animals were used and kept under strictly sterile conditions. Approximately 8 weeks after injection, mice were sacrificed and tumors formed were excised. The specimens were fixed in 4% buffered formalin and embedded in paraffin blocks. Tissues were then cut into 4 μm sections and stained with Haematoxylin–Eosin.

### Microscopy

For light microscopy, samples were fixed either with 1% or 4% formaldehyde in phosphate-buffered saline, permeabilized with 0.2% Triton X-100 and blocked with 0.5% fish skin gelatin according to Maison et al.^88^. Samples were imaged using a Leica SP5 confocal microscope (63x, 100 × magnification lenses, image dimensions 512 × 512, 8-bit), with LAS AF software (Leica Microsystems). Embryoid bodies on Fig. 3a were imaged using Leica DMI6000B microscope (10 × magnification lens), with LAS AF software (Leica Microsystems). All images acquired were further processed using LAS X software (Leica Microsystems).

### RNA extraction

Total RNA from cell culture samples was obtained and purified using the RNeasy Protect mini kit (Qiagen). On-column DNase digestion was performed using an RNase-Free DNase set (Qiagen). Total RNA from fresh mouse tissues was isolated using the PureLink RNA mini kit (Invitrogen) and on-column DNA digestion was carried out using the PureLink DNase set (Invitrogen). RNA quantity of all samples was measured using a NanoDrop Spectrophotometer (Thermo) and its quality was assessed by a Βioanalyzer analysis (Agilent). Only high quality RNA was processed further (RIN value > 9 for cell culture samples and > 7.5 for tissue samples).

### Quantitative real-time PCR

RT-qPCR was performed as described in Karanika et al.^20^. Three biological replicates were used for cell culture samples and four for tissue samples. Based on analysis by the geNorm algorithm (Biogazelle), the CYC1-EIF4A2 and GAPDH-RPL13A gene pairs (PrimerDesign) were selected for normalization of the OE and KO RT-qPCR data, respectively. For tissue samples, the following normalization genes were selected: GAPDH, YWHAZ, ACTB, EIF4A2, UBC, CANX, CYC1 and ATP5B. The primer sequences for each presented gene were as follows:

**Table:** 

Gene Name	Forward primer	Reverse primer
*ms/hu haspin-cells*	ATGCTGAAAAGGTTTATGGGGA	GGCAGGATTTCCTCAAAGGTTT
*Nanog*	CCAGTGGAGTATCCCAGCAT	GTTGGTCCAGGTCTGGTTGT
*Oct4*	TGGGCTAGAGAAGGATGTGG	TGGGAAAGGTGTCCCTGTAG
*Dazl*	GCCCGCAAAAGAAGTCTGTG	ACCAACAACCCCCTGAGATG
*Scml2*	CCACTGGGGTACACACTGAAT	CAGCTCCAATTGTGGCAGGA
*mhaspin-tissues*	GCCGCGTGTAAAAAGGTTGT	GTTCCCGTTGCTTCTTGCTG

Data analysis was carried out by the qbase^+^ analysis software (Biogazelle) according to MIQE guidelines. Statistical analysis in all cases was carried out in qBase^+^, using one way Anova and Tukey–Kramer post-test for the pairwise group comparisons.

### RNA sequencing and bioinformatics analysis

Total RNA from 3 biological replicates in each case was isolated as described above. To generate sequencing libraries TruSeq stranded mRNA library kit (Illumina) was used with 500 ng total RNA as input. The whole protocol was performed on the Biomek FX laboratory automation workstation (Beckman). Libraries (75 bp reads) were sequenced using the NextSeq 500 system (Illumina) at the Genomics Core Facility, EMBL-Heidelberg, Germany. Subsequent analysis of raw data was carried out on the Chipster platform^89^. Initially, FastQC was used to assess data quality and Trimmomatic^90,91^ to perform read trimming. The sequenced reads were aligned using TopHat2 (single end reads, library type: fr-firststrand) on mouse genome GRCm38.87. The aligned reads were counted by HTSeq (union, stranded-reverse) and differential expression analysis was carried out by DeSeq2 (p-value cutoff < 0.05 and |log2 Fold Change| ≥ 1)^92,93^. Gene Ontology analysis was also performed on the Chipster platform with the “GO enrichment for list of genes” tool. Volcano plot visualization and Manhattan distance calculations were performed using the VolcaNoseR software^94^. For a detailed list of deregulated genes, please see Supplementary Data I.

### Frozen sections

All animal experiments and maintenance were carried out in accordance to the institutional and national guidelines for animal experimentation and were ARRIVE compliant (Ethic approval number: EL33-BIOexp01/7491, Directorate for Agricultural Economy and Veterinary Services, Perfecture of Epirus, Greece). Testes and kidneys from 7dpp, 22dpp, 24dpp, 28dpp and adult mice (C57BL/6J) were collected, embedded in OCT compound and snap-frozen in liquid nitrogen. Tissue blocks were sectioned 5 μm thick in a Leica CM 1850 cryotome and processed for indirect immunofluorescence. Frozen sections were fixed with 1% or 4% formaldehyde, blocked with 0.5% fish skin gelatin and 0.2% Triton X-100 according to Maison et al.^88^. Antibodies used are described in Supplemental materials and methods. Meiotically dividing cells in 22dpp and 28dpp mouse testes were identified according to published observations and images^95–97^.

### Histone extraction, immunoprecipitation and western blotting

For total histone extraction, 1 g of testis tissue was decapsulated, thoroughly minced and homogenized (60 strokes on Dounce homogenizer, 3 ml volume capacity). Isotonic buffer containing 20 mM Tris–HCl pH 7.4, 150 mM NaCl, 2 mM MgCl2 and 1 mM PMSF was added (1 ml per 200 mg of tissue) and the homogenate underwent further processing in a larger Dounce homogenizer (15 ml volume capacity, 300 strokes). Triton-X100 was then added to the suspension to a final concentration of 0.5%, followed by 15-min incubation on ice. The cytoplasmic and Triton-X soluble fraction was removed by centrifugation (956*g*/4 °C/5 min) and total histones were extracted from the insoluble residue. To that end, 0.5 N HCl and 10% glycerol (3:1 buffer per pellet volume) were added to the pellet and the suspension was incubated for 30 min on ice. After centrifugation (10,621*g*/4 °C/5 min), the pH was adjusted to 7.5 with the addition of Trizma base crystals and NaCl was adjusted to 300 mM. All procedures were carried out on ice, in the presence of phosphatase inhibitors (20 mM β-glycerol phosphate, 50 mM NaF, 50 mM sodium orthovanadate, all from Sigma-Aldrich). For immunoprecipitation experiments, histone samples were dialyzed overnight at 4 °C against buffer A (50 mM Tris–HCl pH 7.4, 300 mM NaCl, 1 mM EGTA, 1 mM PMSF), collected and cleared by centrifugation (20,817 g/4 °C/15 min). For blocking purposes, Tween-20 and BSA were added to a final concentration of 0.01% and 0.5%, respectively, to each sample. Subsequently, the samples were incubated with primary antibodies for 3.5 h at 4 °C. Protein G Sepharose beads (Protein G Sepharose 4 Fast Flow, GE Healthcare, 17-0618-01) were equilibrated with buffer A containing also 0.01% Tween-20 and 0.5% BSA, for 2 h at 4 °C. The sample-antibody mixtures were added to the beads and incubated for 1 h at 4 °C. Beads were then washed with buffers B (50 mM Tris–HCl pH 7.4, 150 mM NaCl, 1 mM EGTA, 1 mM PMSF and 0.5% Triton X-100) and C (50 mM Tris–HCl pH 7.4, 300 mM NaCl, 1 mM EGTA, 1 mM), in the presence of phosphatase inhibitors. Samples were analyzed by SDS-PAGE. Western blotting was carried out according to standard procedures. For full length gels and blots, see Supplemental Data III.

## Supplementary Information

Supplementary Information 1.

Supplementary Information 2.

Supplementary Information 3.

Supplementary Information 4.

## Data Availability

RNA-Seq data have been deposited in NCBI's Gene Expression Omnibus (GEO) and are accessible through GEO Series accession number GSE154246.
