# Supplementary material for: Distinct roles of haspin in stem cell division and male gametogenesis
Source: Sci Rep. 2021 Oct 6;11:19901. doi: 10.1038/s41598-021-99307-8 (PMC8494884; doi:10.1038/s41598-021-99307-8)
Supplement: Supplementary file 3 — Supplementary Information 3. [file 41598_2021_99307_MOESM3_ESM.pdf]

Supplementary data III

Fig. SD1

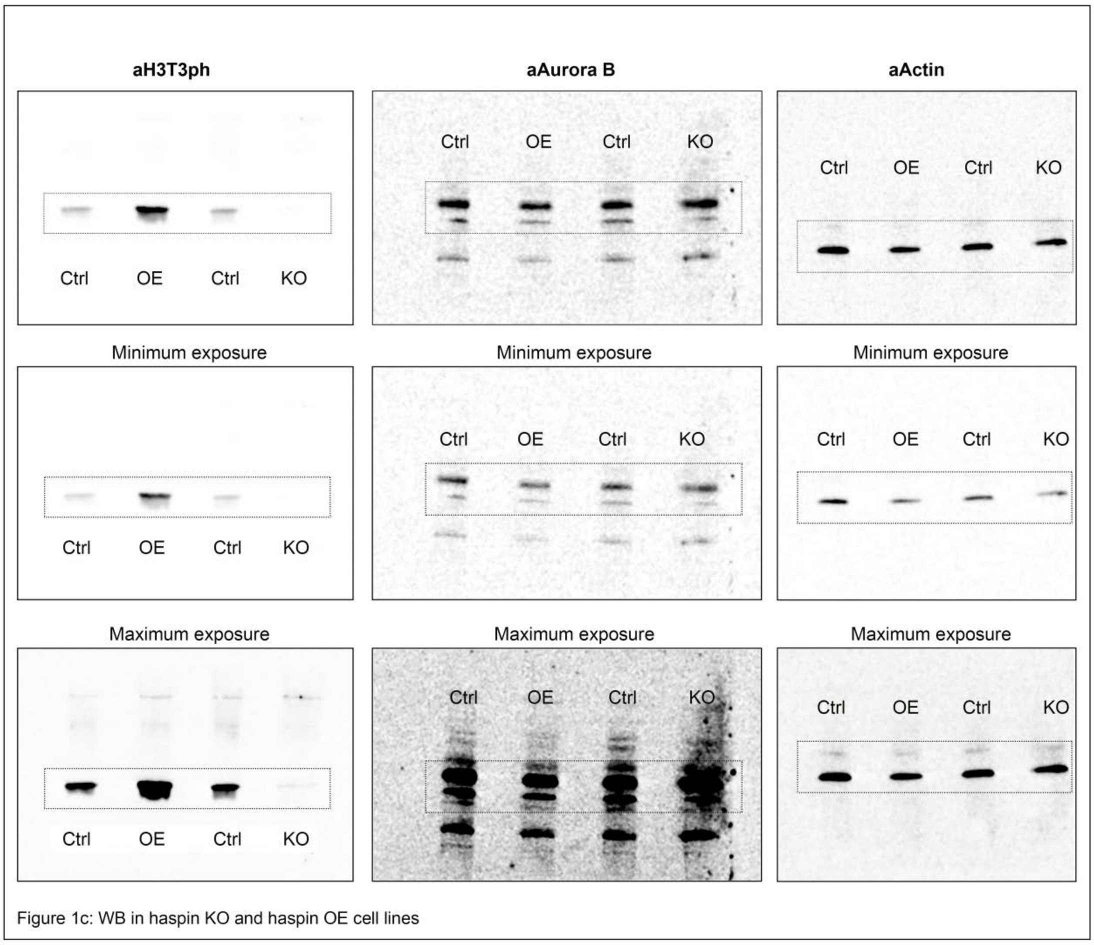

Figure SD1. Raw data used in Figure 1c of the main manuscript. The figure depicts the nitrocellulose filters propped with the corresponding antibodies, after processing in the Azure 600 imaging system. The minimum and maximum exposure images are included in each case. The parts of the immunoblots shown in Figure 1c are boxed. Control and haspin KO/OE clones are marked in the filters along with the antibodies used in each case (top).

Fig. SD2

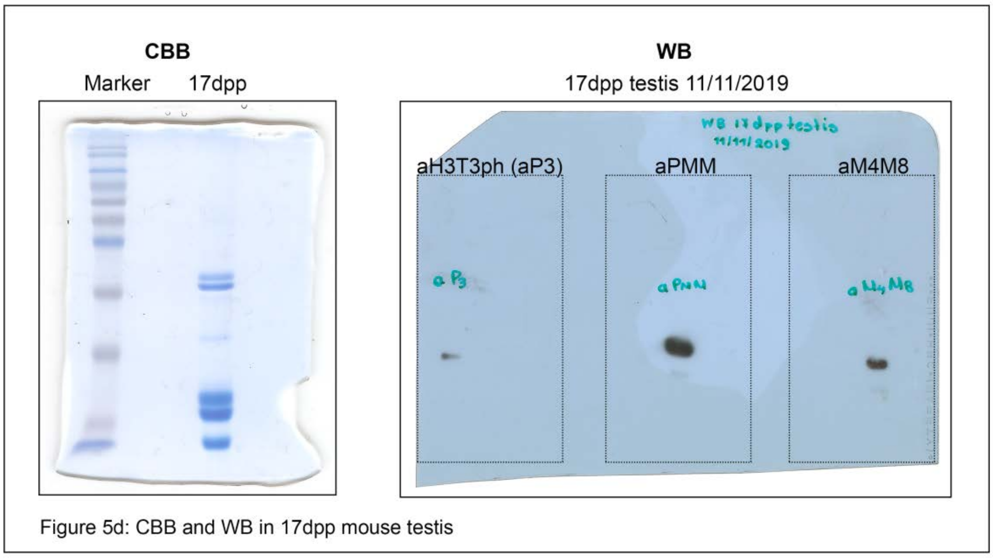

Figure SD2. Raw data of 17dpp testis used in Figure 5d of the main manuscript. The figure depicts the Coomassie Brilliant Blue (CBB) stained gel (left) and an ECL film (right, *WB*), with the three nitrocellulose filters. The parts of the data shown in Figure 5d are boxed.

Fig. SD3

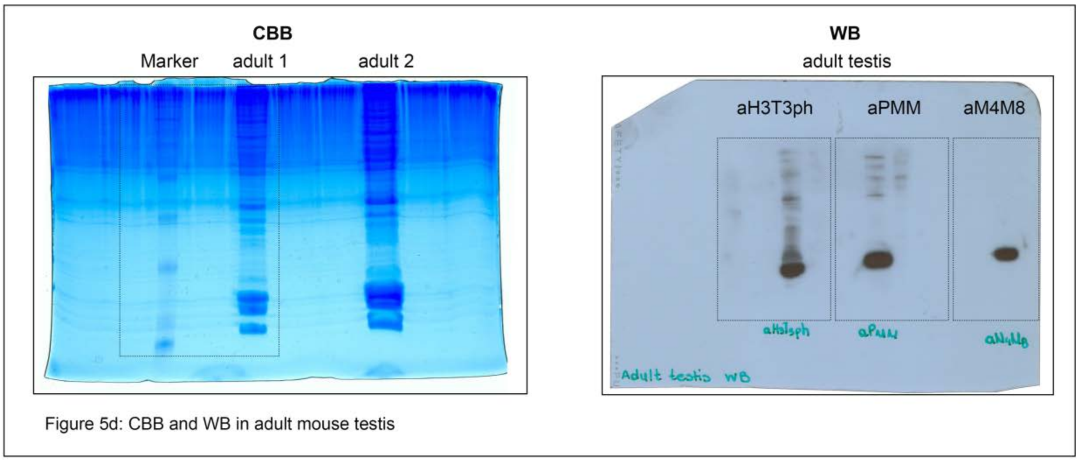

Figure SD3. Raw data of adult testis used in Figure 5d of the main manuscript. The figure depicts the Coomassie Brilliant Blue (CBB) stained gel (left) and an ECL film (right, *WB*), with the three nitrocellulose filters. The parts of the data shown in Figure 5d are boxed.

Fig. SD4

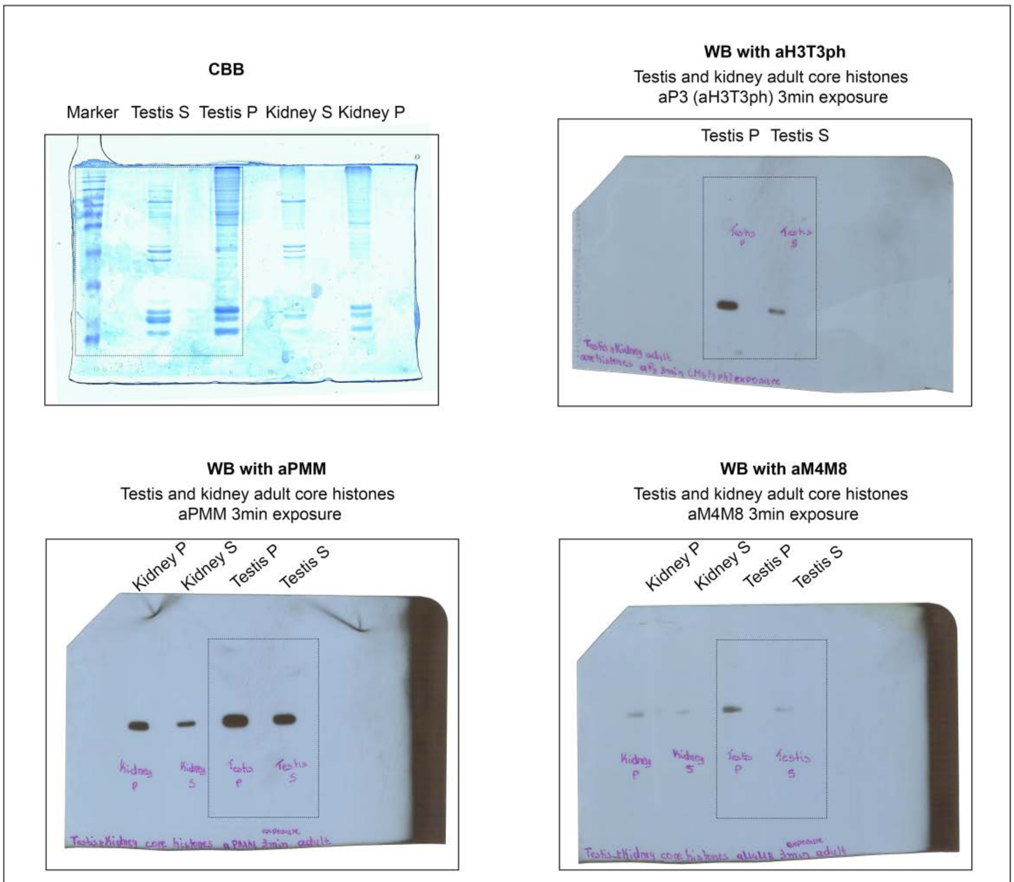

Figure 5d: CBB and WB in S, P samples from adult mouse testis

Figure SD4. Raw data of the adult testis samples used in Figure 5d of the main manuscript. The figure depicts the Coomassie Brilliant Blue (CBB) stained gel (upper left) and three ECL films with the corresponding nitrocellulose membranes (upper right and lower images, *WB*). The parts of the immunoblots shown in Figure 5d are

boxed. S corresponds to a high-speed supernatant of the tissue extract and P to the pellet.

Fig. SD5

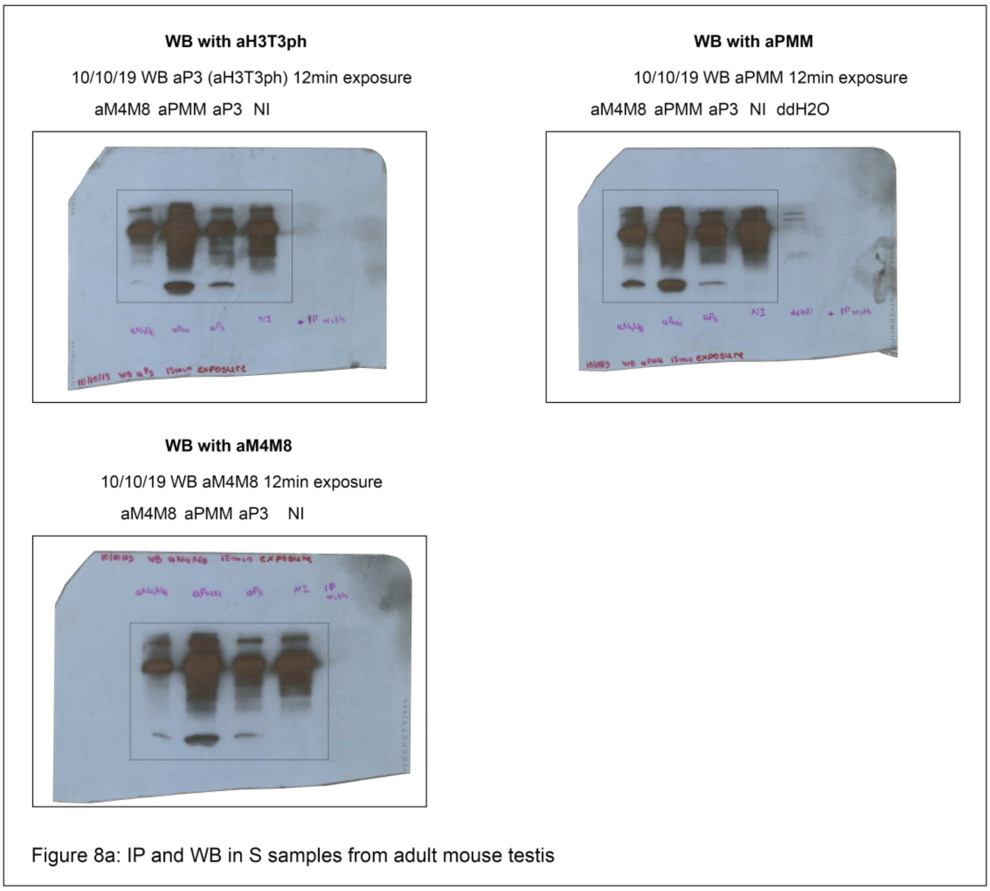

Figure SD5. Raw data of the adult testis samples used in Figure 8a of the main manuscript. The figure depicts three ECL films with the corresponding nitrocellulose membranes, after the immunoprecipitation experiments. The parts of the immunoblots shown in Figure 8a are boxed and the antibodies used in each case are marked on top.
