# Supplementary material for: Distinct roles of haspin in stem cell division and male gametogenesis
Source: Sci Rep. 2021 Oct 6;11:19901. doi: 10.1038/s41598-021-99307-8 (PMC8494884; doi:10.1038/s41598-021-99307-8)
Supplement: Supplementary file 4 — Supplementary Information 4. [file 41598_2021_99307_MOESM4_ESM.pdf]

Supplemental materials and methods

Live cell imaging

Control and haspin cell lines (KO/OE) were transfected with H2B-mCherry in order to enable visualization during M phase. 24 hours post-transfection live-cell imaging was performed using the Incucyte ZOOM system, with a 20x magnification lens. Cells were monitored for 48 hours and images were acquired every 15 minutes. The timed images were used to track the number of cells completing mitosis and the duration of the division for each case.

FACS experiments

For cell cycle analysis, 10^6 cells were processed using a BD FacsCalibur Flow Cytometer after staining with 50 ng/ml Propidium Iodide for 30 min, as described in Vartholomatos et al.[1]. Data were processed with CellQuest v.3.1 software.

Antibodies

The following primary antibodies were used: anti-H3T3ph (dilution 1:750), anti-PMM (dilution 1:600), anti-M4M8 (1: 100), all raised in house (Polioudaki et al., 2004[10]; Markaki et al., 2009[8]); anti-H3K9me$_3$ (dilution 1:200), donated by P. B. Singh, Charité- Universitätsmedizin (Germany) and one from Novus Biologicals (NBP1-30141; dilution 1:800); anti-H3S10ph (ab14955; dilution 1:1,000 for cultured cells and 1:2,000 for tissue sections), anti-Aurora B (ab2254; dilution 1:1,000 for cultured cells and 1:250 for tissue sections), anti-Nanog (ab80892; dilution 1:300 for immunofluorescence) all purchased from Abcam, UK; anti-

$H3K27me_3$ (Upstate, 07449; dilution 1:400); anti-α-tubulin (Sigma-Aldrich; T5168, dilution 1:5,000), anti-Actin (C-4, Millipore, mab1501, dilution 1:1000); anti-γH2AxS139ph (Millipore 05-636-I; dilution 1:1,500); anti-Oct3/4 (Santa Cruz, sc-5279; dilution 1:300), anti-Klf4 (Abcam, ab129473, dilution 1:1000 for immunofluorescence), anti-HP1α (Millipore, MA3584; dilution 1:1,000 for cultured cells and 1:600 for tissue sections); anti-SCML2, a gift from Dr. Namekawa[33] (dilution 1:1,500); anti-INCENP (Active Motif, 39259; dilution 1:500); ACA, a gift from A. Tzioufas, University of Athens, Greece (dilution 1:300); anti-H2AT120ph (Active Motif, 61195; dilution 1:500) and anti-DAZL (Santa Cruz, sc-390929; dilution1:50 and Millipore, ABD 31; dilution 1:100). The following secondary antibodies were used: goat anti-rabbit 488 (Alexa, A11008), goat anti-rabbit 568 (Alexa, A11011), goat anti- mouse 569 (Alexa, A11004), goat anti-human 568 (Alexa, A21090) and goat anti-rabbit HRP (Invitrogen, 65-6120). DNA was stained with TO-PRO 3 (Invitrogen, T3605; dilution 1:5,000).

Amplicon Sequencing

Amplicon Sequencing was performed as described in Karanika et al.[20].

Competitive inhibition assays

Frozen tissue sections were examined by indirect immunofluorescence as described in Supplemental materials and methods. Primary antibodies were pre-incubated with either the control (unmodified) or the H3-modified peptides in blocking buffer, for 15 min, at room temperature. Quantification of the signal intensity at the level of single cells was performed using the Image J software. For this purpose, four confocal sections in each tubule were projected onto one another

and the maximum intensity for each cell was measured using the circular tool. Peptides used for competition experiments were synthesized and HPLC-purified at the Rockefeller University Proteomics Resource Center. The exact sequences and concentrations per experiment are as follows:

**Name**	**Sequence**	**Figure & Concentration**
H3 unmodified	ARTKQTARKSTGGKAPRKQC	5D 1μM, 6C & 6D 0.5μM
H3T3ph	ART(ph)KQTARKSTGGKAPRKQC	5D 1μM, 6C & 6D 0.5μM
H3K4me$_3$	ARTK(me$_3$)QTARKSTGGKAPRKQC	0.5μM
H3R8me$_2$	ARTKQTAR(asym-me$_2$)KSTGGKAPRKQC	0.5μM
H3T3phK4me$_3$R8me$_2$	ART(ph)K(me$_3$)QTAR(asym-me$_2$)KSTGGKAPRKQC	0.5μM
H3K4me$_3$R8me$_2$	ARTK(me$_3$)QTAR(asym-me$_2$)KSTGGKAPRKQC	0.5μM
H3T3phK4me$_3$	ART(ph)K(me$_3$)QTARKSTGGKAPRKQC	0.5μM
H3T3phR8me$_2$	ART(ph)KQTAR(asym-me$_2$)KSTGGKAPRKQC	0.5μM

Statistics

All statistical analysis, unless stated otherwise, was performed using the SPSS statistical package. The exact tests used were selected based on the nature of the data to be compared. Where possible, p-values were depicted with asterisks. More specifically, one asterisk corresponds to $p < 0.05$, two asterisks to $p \leq 0.01$ and three asterisk to a $p \leq 0.001$. For exact number of experiments performed and relevant p-values obtained see Supplementary Data II.

Words: 563

Supplemental Figures

Fig. S1

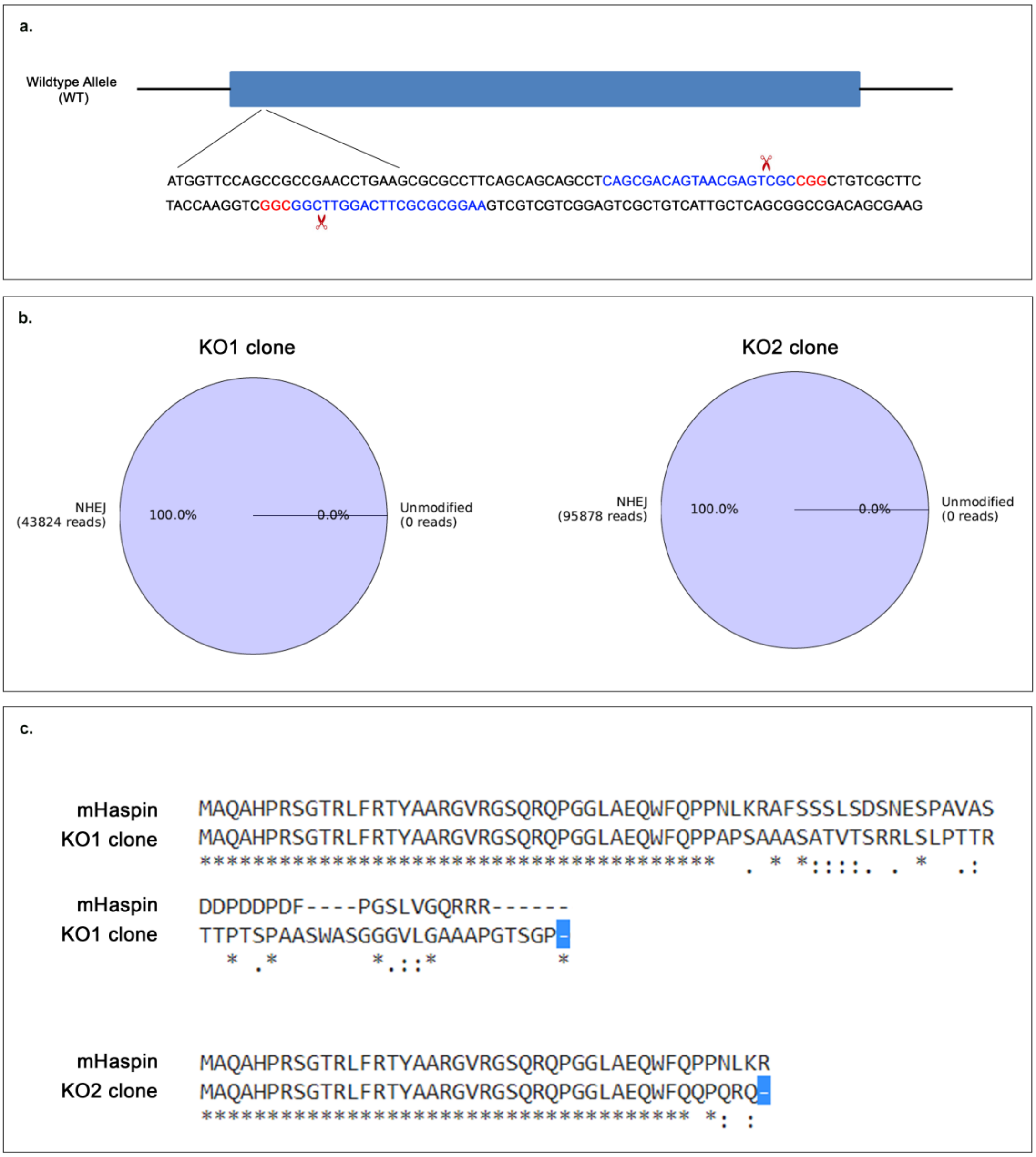

Figure S1. (a) Guide RNAs used to create the haspin-KO clones, as indicated on the genomic mouse haspin sequence (Blue-20nt gRNAs, Red-PAM sequences). (b) Pie diagram showing the NHEJ (Non-Homologous End Joining) frequency of haspin-KO clones 1 and 2, as calculated by the CRISPResso algorithm. (c) Sequence alignment

showing how the amino acid sequence is altered in the two haspin-KO clones. Stop codons are highlighted. See also Karanika et al. [20].

Fig. S2

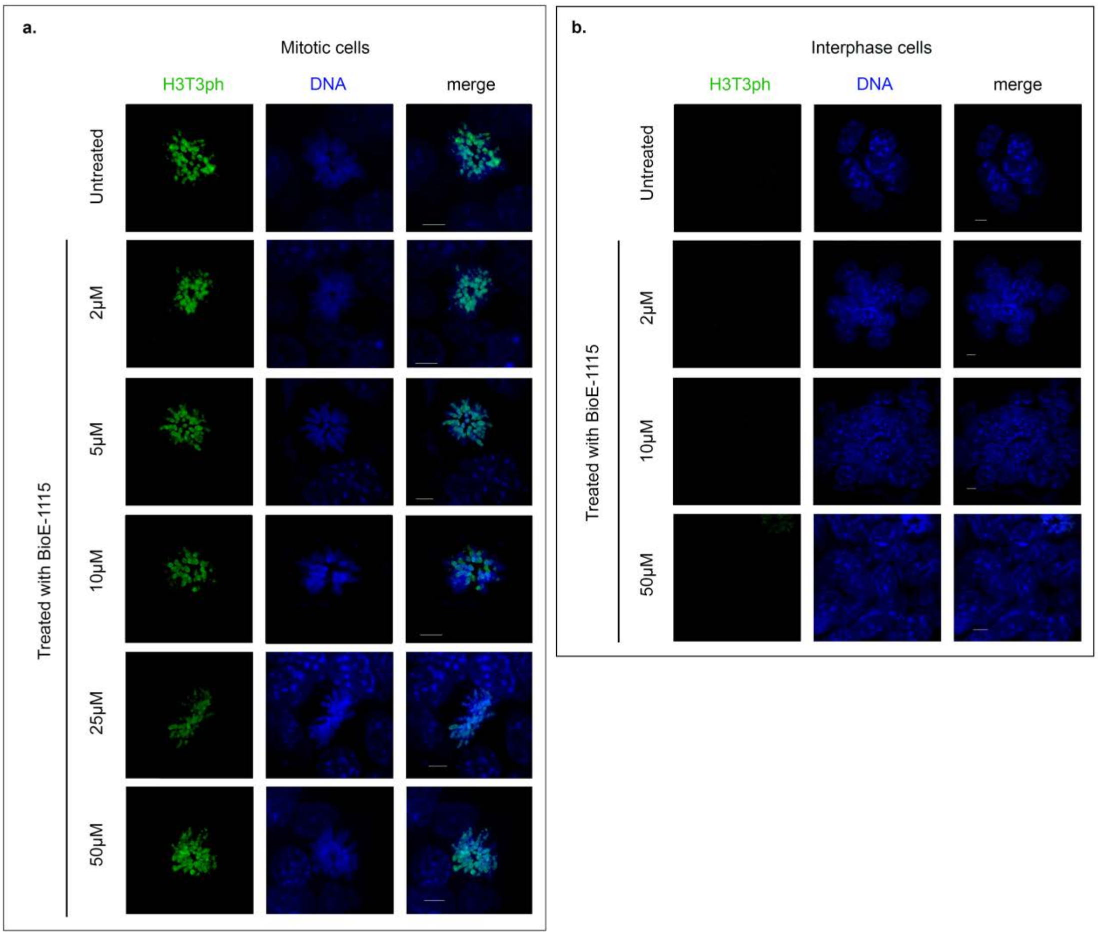

Figure S2. Staining of metaphase (a) and interphase (b) E14 cells for H3T3ph after treatment with various concentrations of the PASK inhibitor BioE-1115. DNA has been stained with TO-PRO 3. Scale bars, 5μm.

Fig. S3

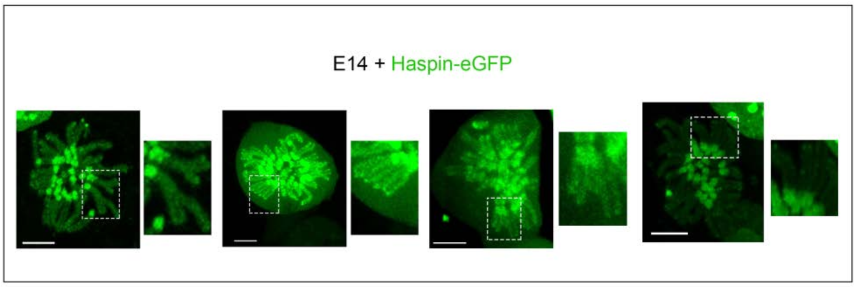

Figure S3. Distribution of haspin-eGFP on metaphase chromosomes in transiently transfected E14 cells. Blowups on the right of each image (at higher contrast) show in detail the dissociation of sister-chromatids. Scale bars, 5μm.

Fig. S4

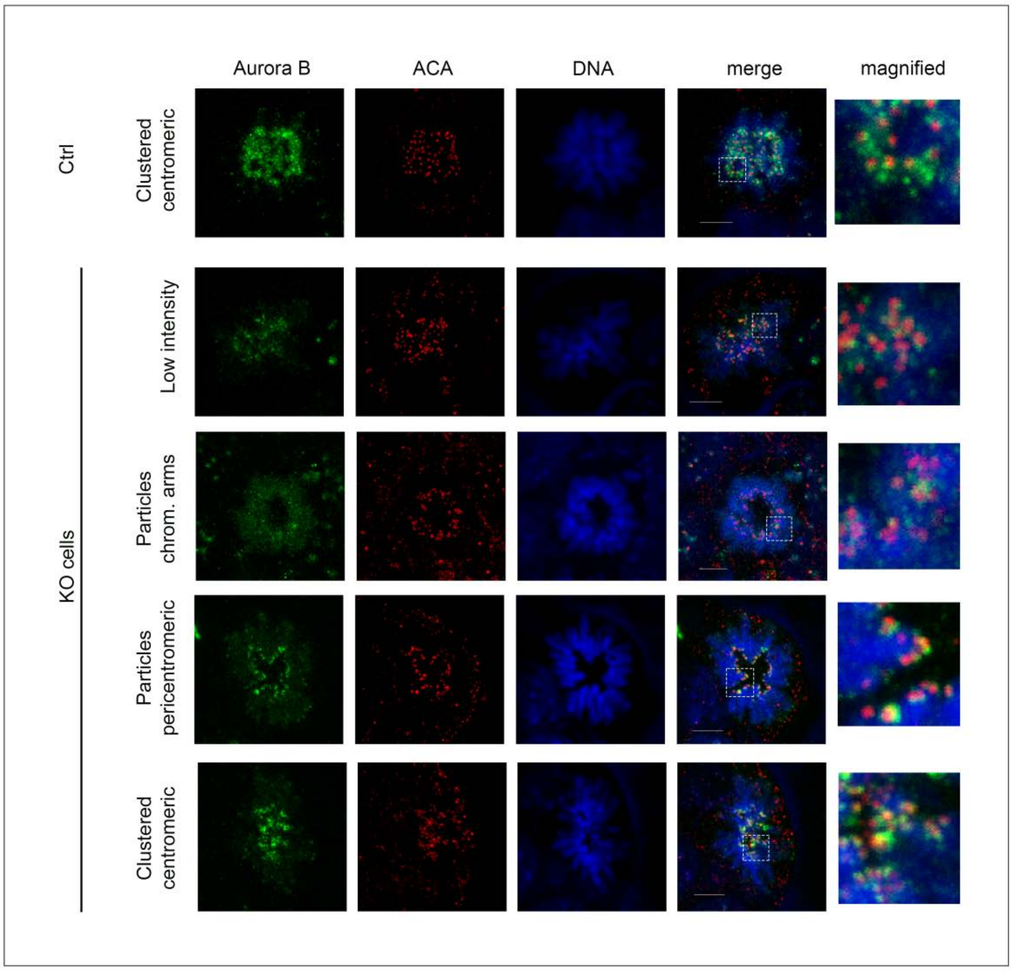

Figure S4. Representative images of E14 cells stained for Aurora B. The images depict different patterns of Aurora B localization in metaphase. These patterns are detected in both control and genetically manipulated cells (KO/OE), albeit in different proportions (see Fig. 2). DNA has been stained with TO-PRO 3. Scale bars, 5μm.

Fig. S5

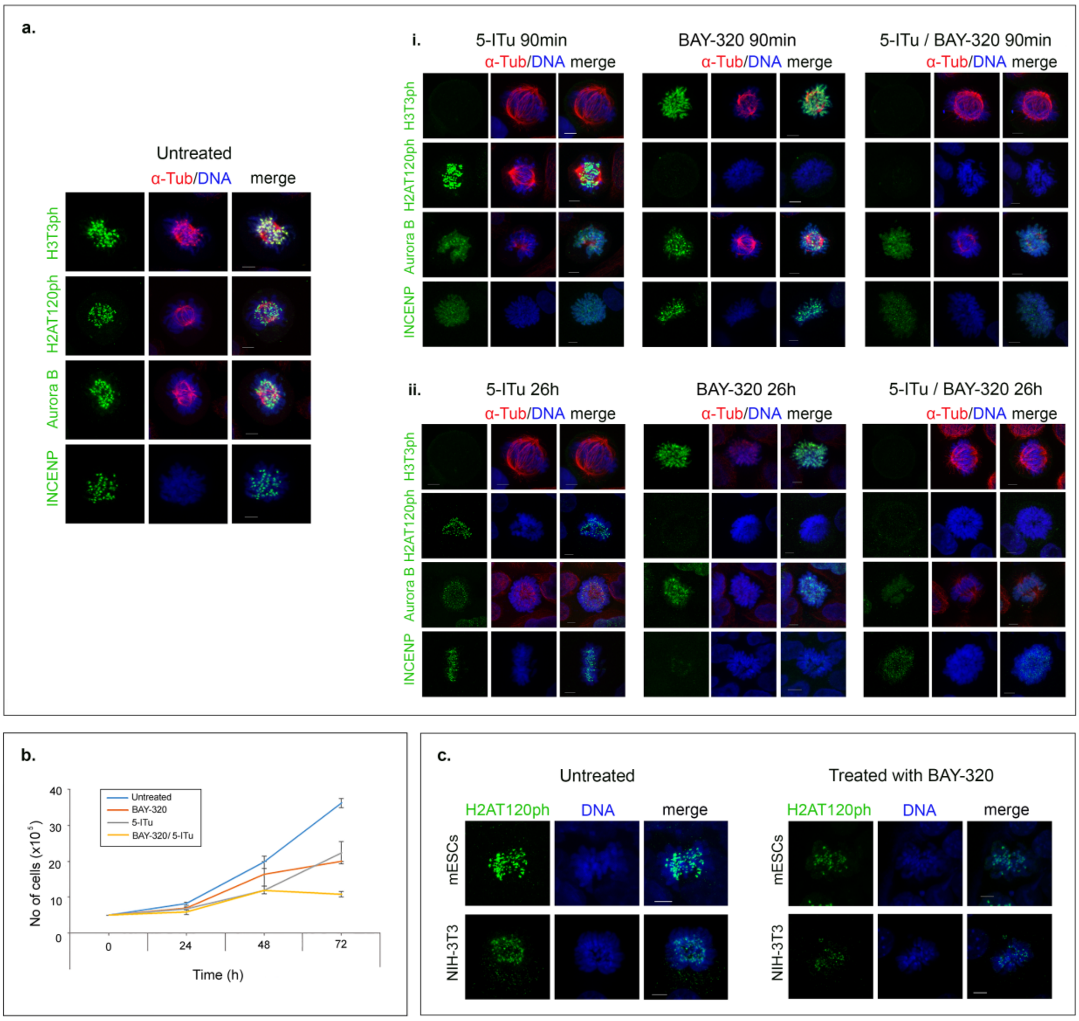

Figure S5. (a) Staining of Hela cells for H3T3ph, H2AT120ph, Aurora B and INCENP after a 90-minute (i) or a 26-hour-(ii) treatment with the haspin inhibitor 5-

ITu (1µM), the Bub1 inhibitor BAY-320 (3µM), and a combination of the two. DNA has been stained with TO-PRO 3. (b) Growth curves of treated (5-ITu: 1µM, BAY-320: 3µM or combination of the two) and untreated control cells. (c) Staining of mouse E14 cells and mouse fibroblasts (NIH-3T3) with a-H2AT120ph before and after treatment with BAY-320 (3µM, 26hrs). Scale bars, 5µm.

Fig. S6

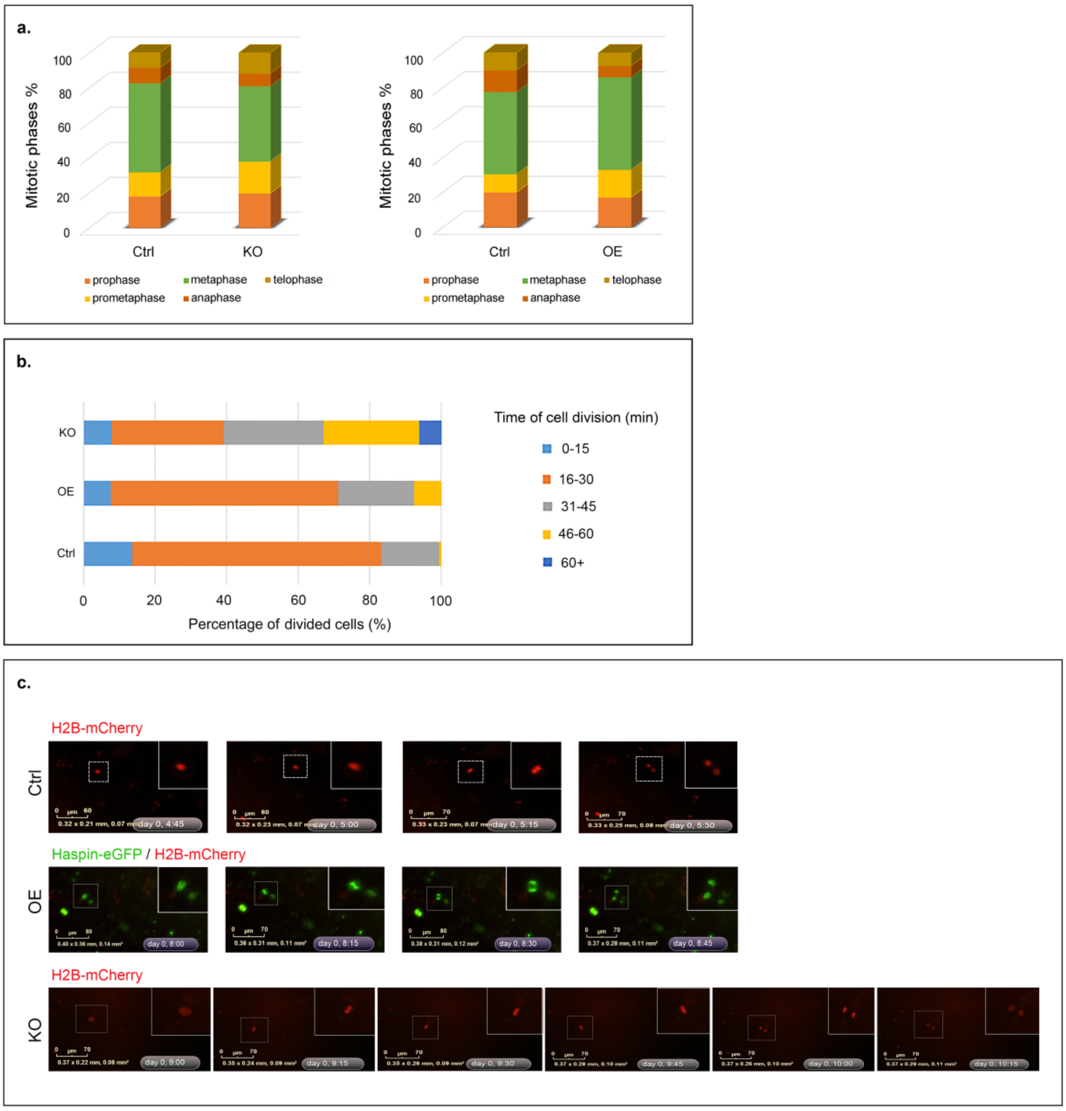

Figure S6. (a) Distribution of haspin-KO and haspin-OE cells in the various phases of mitosis. For quantitative details see Supplementary Data II. (b) Histogram depicting the percentage of cells (control, haspin-KO and haspin-OE) completing mitotic division, according to its duration (min). For quantitative details see Supplementary Data II. (c) Representative images from time-lapse videos of control, haspin-KO and haspin-OE cells undergoing mitosis. Chromatin of distinct cells was detected by H2B-mCherry fusion protein. Blow-up images show details of dividing cells.

Fig. S7

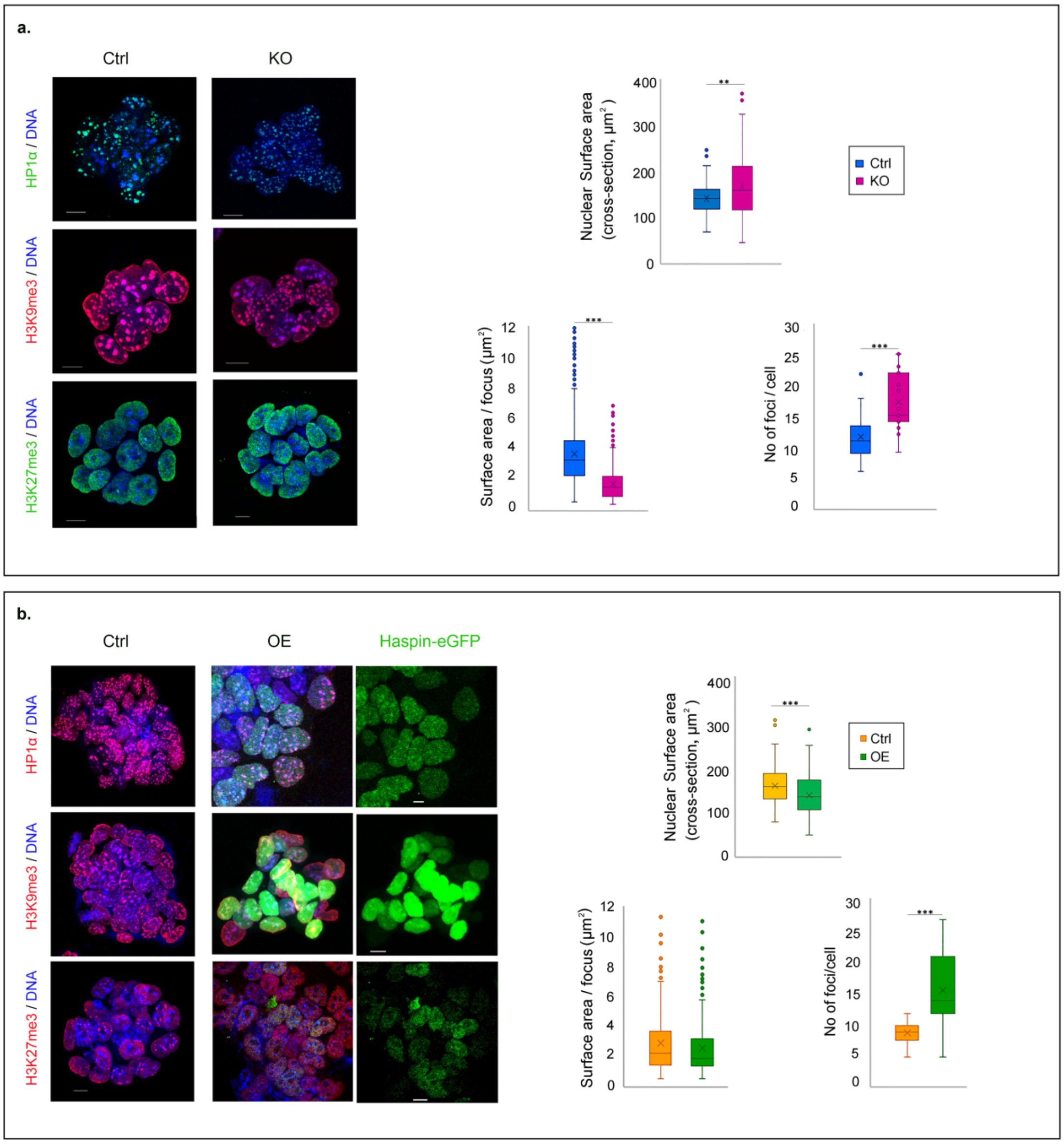

Figure S7. Staining of haspin-KO (a) and OE (b) cells with antibodies against HP1α (Heterochromatin Protein 1α), H3K9me3 (histone H3 tri-methylated at lysine 9) and H3K27me3 (histone H3 tri-methylated at lysine 27). Profiles of the corresponding controls are also included. The boxplots display differences in nuclear surface area (*top*), size (*bottom*, *left*) and number (*bottom*, *right*) of heterochromatic foci in haspin-KO (a) and haspin-OE (b) cells, as compared to controls. Scale bars, 10µm.

Surface area has been measured using Fiji software. For quantitative details, see Supplementary Data II.

Fig. S8

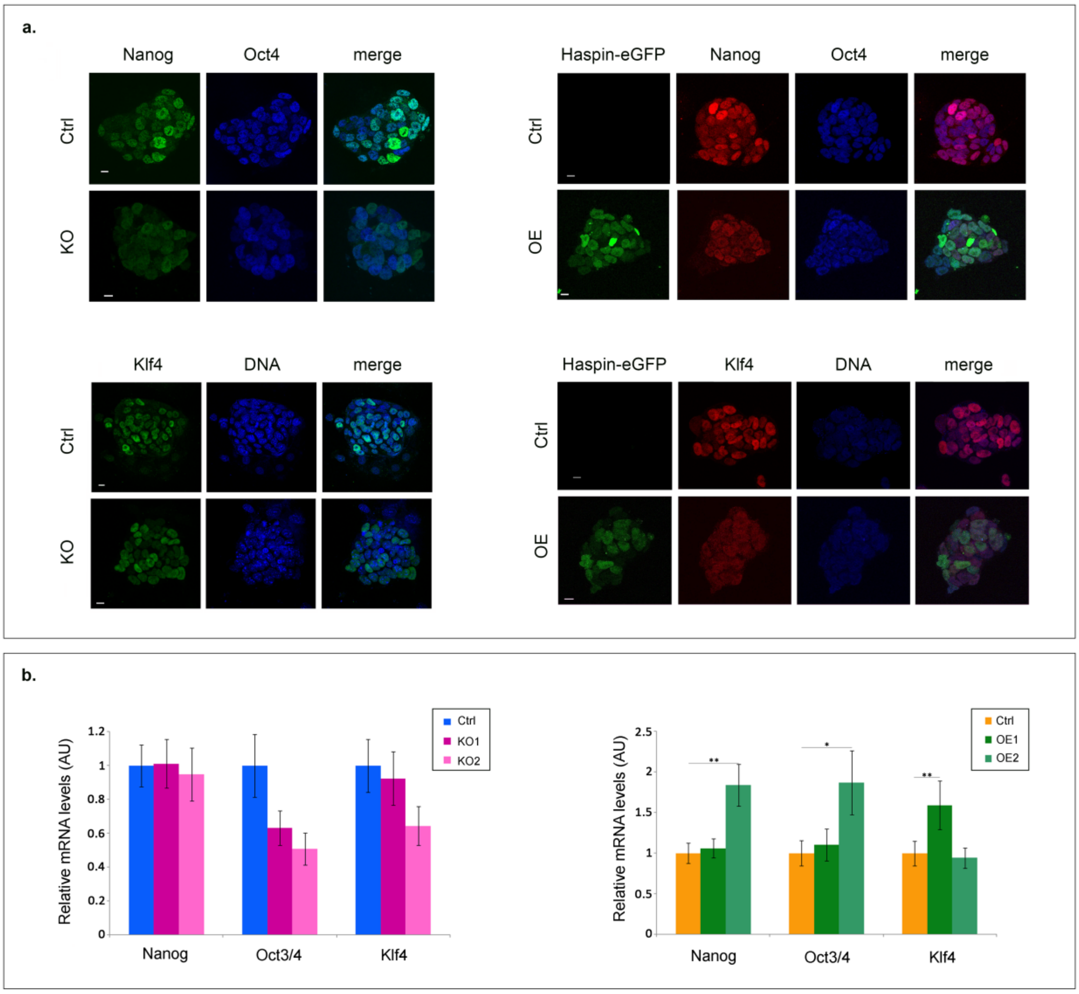

Figure S8. (a) Profiles of haspin-KO (*left*), haspin-OE (*right*) cells and their respective controls stained with antibodies against Nanog, Oct4 and Klf4. Scale bars, 10 µM. (b) Expression levels of Nanog, Oct4 and Klf4 in haspin-KO (*left*) and OE (*right*) cells measured by RT-qPCR. Relative mRNA levels are shown.

Fig. S9

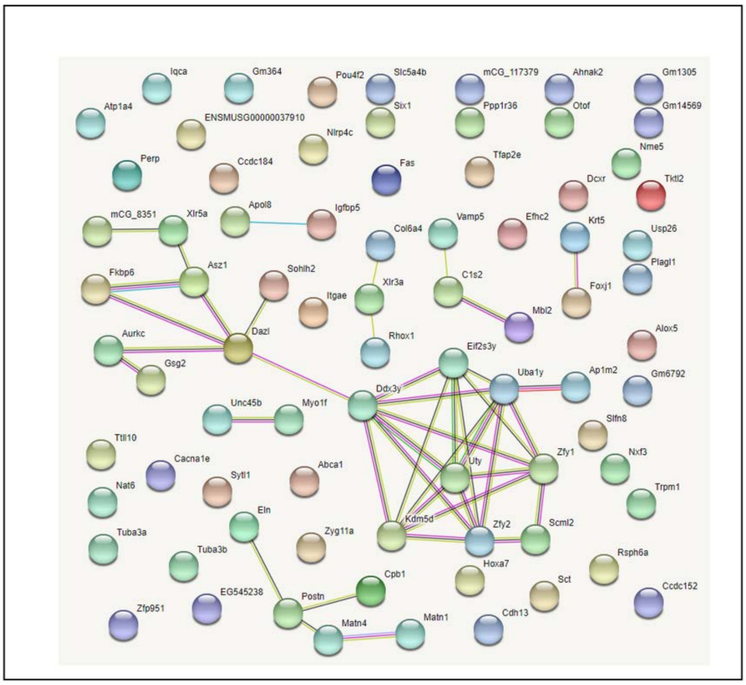

Figure S9. A protein-protein interaction network showing the correlation between deregulated genes in haspin-KO cells with a |$\log_2$ Fold Change| ≥0.95. The network has been constructed using the STRING tool and database. The nodes represent proteins and the edges predicted functional associations. Edges are drawn with differently colored lines, which correspond to types of evidence used in predicting the associations (for more information see Szklarczyk et al., 2019 [59]).

Fig. S10

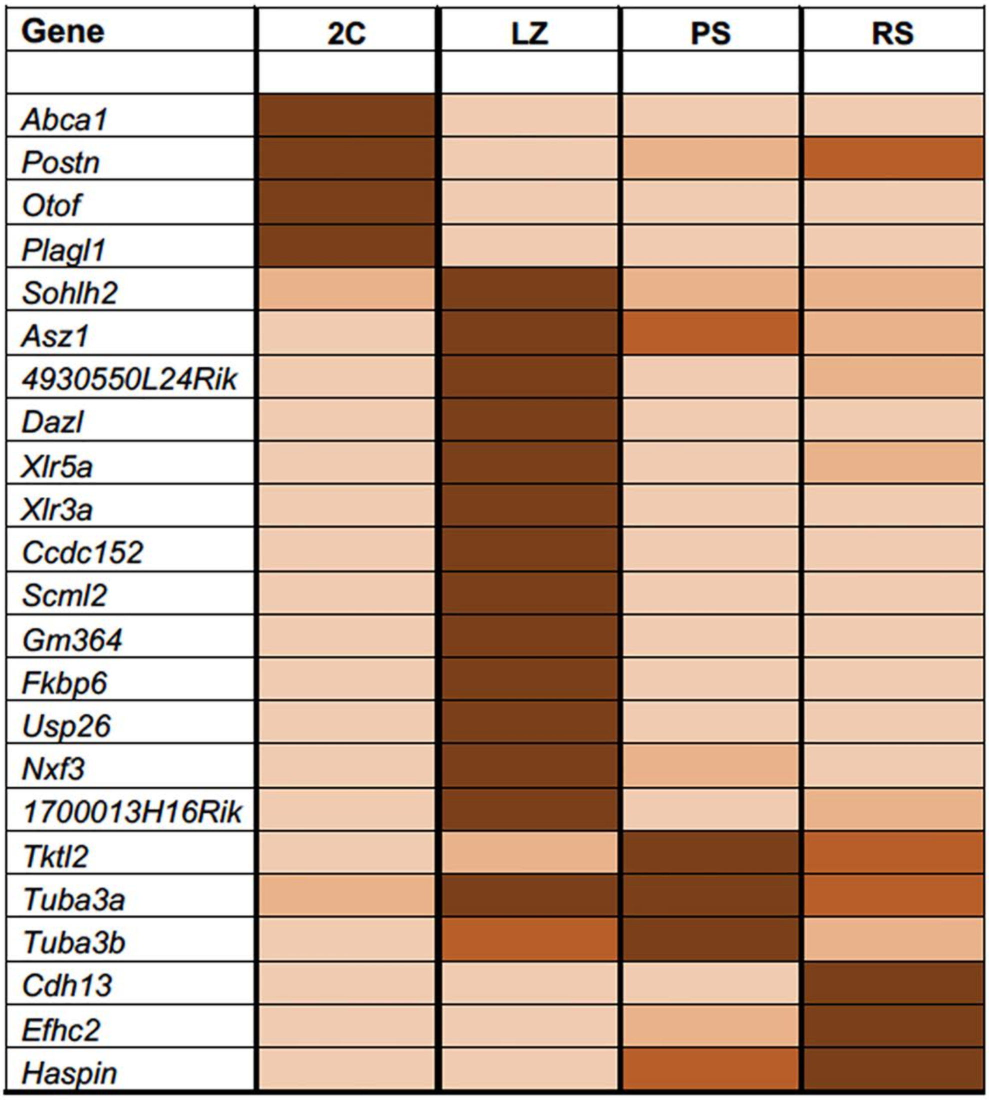

Figure S10. Expression levels of genes downregulated in both haspin KO and OE cells during male gametogenesis. The list depicts in shades of brown (from low to high) the relative density of transcripts (RPKM values) for 22 of the genes identified in our screen, as previously determined by da Cruz et al. after analysis of sorted testicular cells[65]. Apparent in the list are genes whose expression peaks before meiosis (2C), at the leptotene-zygotene (LZ), the pachytene (PS) or the round spermatid (RS) stage.

Fig. S11

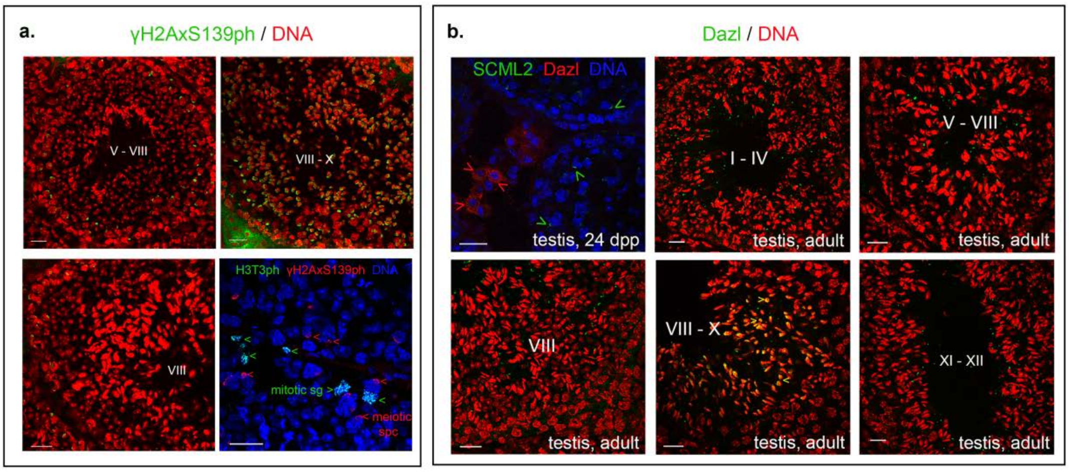

Figure S11. (a) Occurrence of the meiotic recombination mark γH2AxS139ph through the seminiferous cycle in comparison to the H3T3ph mark, as revealed after staining with the corresponding antibodies (*mitotic sg*: mitotic spermatogonia; *meiotic spc*: meiotic spermatocytes). (b) Localization pattern of DAZL in the seminiferous tubules of immature and mature mouse testes, as detected by indirect immunofluorescence. The antigen is localized in the cytoplasm of immature spermatogonia and in the nuclei of late round and elongating spermatids. DNA has been stained with TO-PRO 3. Scale bars, 20μm. Latin numerals within the images indicate the stage of each tubule.

Fig. S12

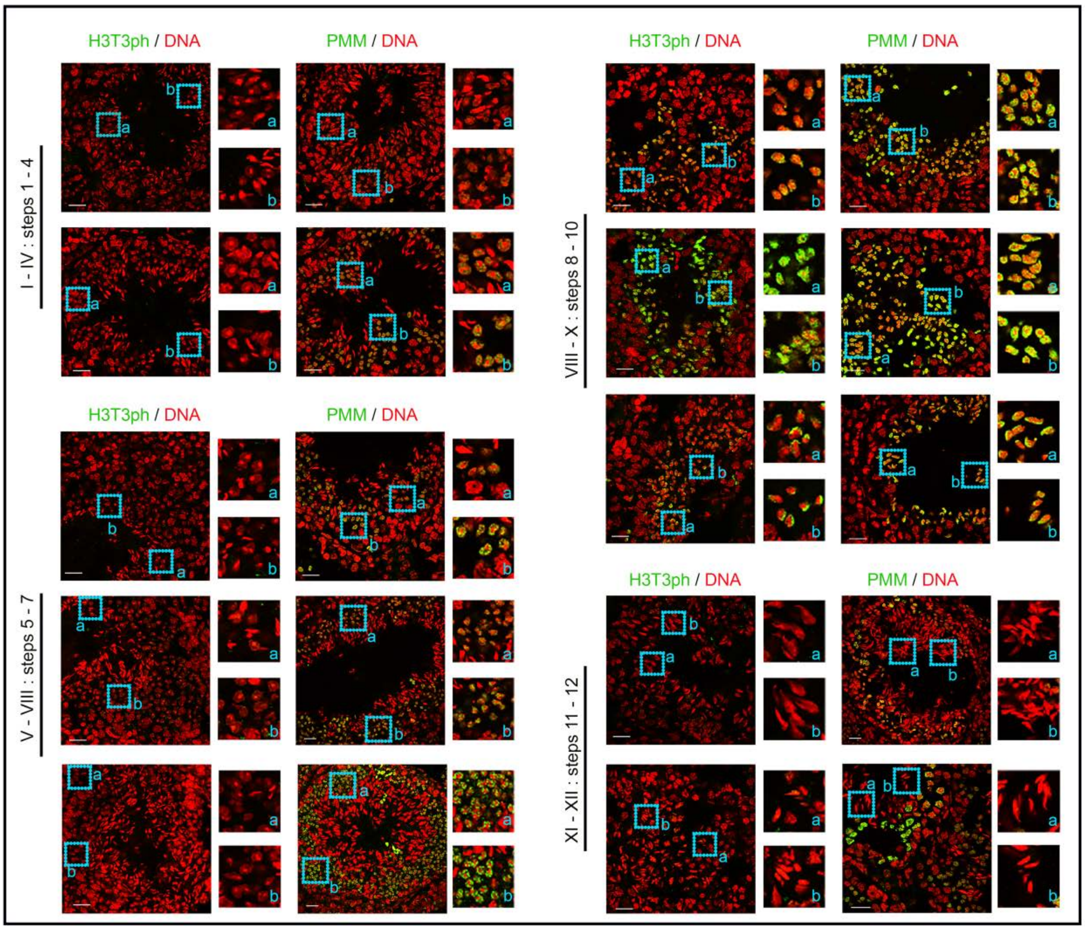

Figure S12. Establishment and removal of the H3T3ph and PMM marks during the seminiferous cycle after staining sections of mature mouse testes with the corresponding antibodies. DNA has been stained with TO-PRO 3. Scale bars, 20µm. The stage of each tubule and the steps of spermiogenesis are indicated by Latin and Arabic numerals, respectively.

Fig. S13

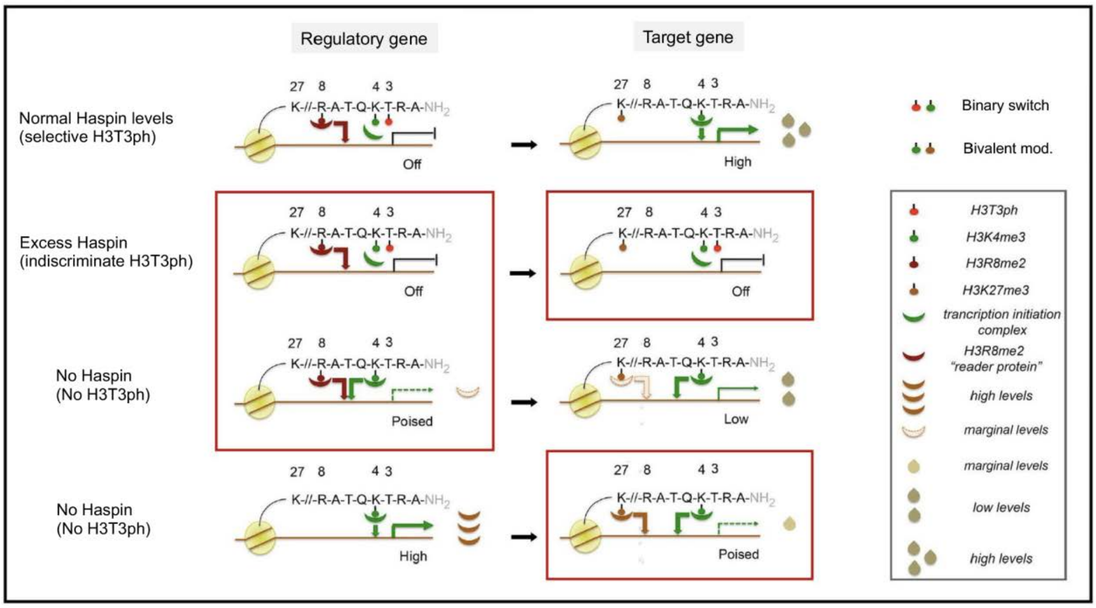

Figure S13. Tentative model of haspin's function based on a compound phospho-methyl switch. For details see *Discussion*.

Reference list

1. Vartholomatos, G. et al. Rapid cell cycle analysis for intraoperative diagnosis of brain tumors. *Brain Tumor Pathol*. **32**(2), 151-152 (2015). doi: 10.1007/s10014-014-0201-5.
